# Lineage specific conservation of cis-regulatory elements in Cytokinin Response Factors

**DOI:** 10.1038/s41598-019-49741-6

**Published:** 2019-09-16

**Authors:** Rachel V. Powell, Cipher R. Willett, Leslie R. Goertzen, Aaron M. Rashotte

**Affiliations:** 101 Rouse Life Sciences, Department of Biological Sciences, Auburn University, Auburn, AL 36849 USA

**Keywords:** Plant evolution, Plant genetics

## Abstract

Expression patterns of genes are controlled by short regions of DNA in promoter regions known as cis-regulatory elements. How expression patterns change due to alterations in cis-regulatory elements in the context of gene duplication are not well studied in plants. Over 300 promoter sequences from a small, well-conserved family of plant transcription factors known as Cytokinin Response Factors (CRFs) were examined for conserved motifs across several known clades present in Angiosperms. General CRF and lineage specific motifs were identified. Once identified, significantly enriched motifs were then compared to known transcription factor binding sites to elucidate potential functional roles. Additionally, presence of similar motifs shows that levels of conservation exist between different CRFs across land plants, likely occurring through processes of neo- or sub-functionalization. Furthermore, significant patterns of motif conservation are seen within and between CRF clades suggesting cis-regulatory regions have been conserved throughout CRF evolution.

## Introduction

As master regulators, transcription factor (TF) proteins can bind to many different gene targets allowing plants to spatiotemporally control gene expression as well as entire regulatory cascades. This regulatory control serves to help plants adapt to their environment^1^. TF proteins function by binding to specific short DNA base pair patterns called motifs or cis-regulatory elements (CREs) in upstream, intron, or downstream regions of target genes. In order to physically bind TFs, a chemical interaction between the amino acid side chain of TF proteins and the CREs occurs, causing binding and subsequent effects of the TF^2^. In many instances, a particular DNA motif is able bind more than one TF, with differing responses^3^.

Plant CREs are incredibly specific allowing for distinct and differential control of gene expression dependent upon the life stage, location, and environmental conditions. Regulation of transcription is dependent upon both presence of TFs and number, location, and specific combinations of CREs present in the promoter region of any specific gene^4^. Additionally, TF binding is dependent upon cell type and combinatorial effects of additional TFs, co-factors, and chromatin state^2,5^. Every upstream promoter sequence contains three main regions: the core, proximal, and distal promoters. The core promoter contains the well-known TATA box, but a TF can bind hundreds to thousands of bp away from the TSS, in the proximal or distal regions, and still influence transcription.

Since the discovery of CREs, there has been much debate about whether the linear order of CREs in the promoter plays a critical role in affecting gene function. Additionally, the location or distance of CREs relative to the gene they are regulating can also be a defining characteristic for transcript regulation. When binding sites are in close proximity, TFs can work in conjunction with other bound TFs to bolster affects^2^. However, there are many differing opinions on whether CREs position and orientation is more important than the simple presence of all the necessary binding sites^4,6,7^. Regardless, a majority (86%) of all *A. thaliana* TF binding sites were found to be located from 1000 bp upstream of the TSS to 200 bp downstream from the end of the coding region^8^.

As organisms evolve, their genomes are not static. Instead, genes and even whole genomes are known to duplicate, particularly in plants. When genes duplicate, there are four different potential fates for the protein created from coding sequences: neofunctionalization, subfunctionalization, pseudogene, and conservation of original function. However, the level of conservation within cis-regulatory regions remain unclear. Alterations in exonic gene coding sequences can have severe effects, such as a premature stop codon, but alterations in promoter sequences tend to be less severe in nature, but can lead to different spatiotemporal expression between these duplicated genes, which is described as the first step in functional differentiation^8,9^. As mutations accumulate, phenotypic changes appear between the once identical duplicated genes leading to neo- or sub-functionalization, allowing genes to become unique and function during certain life stages or plant tissues^9^. Therefore, a primary driver of evolution is the alteration of CREs, resulting in expression divergence between duplicated genes^10–12^.

**Figure 1:**
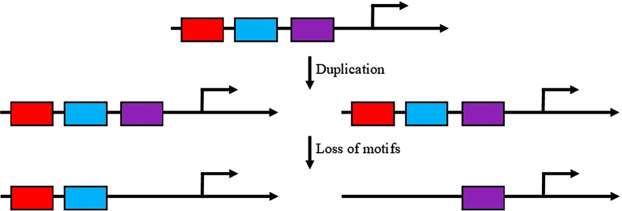
The DDC (divergence-deletion-complementation) model of cis-regulatory element CRE evolution. After a gene duplicates, the two copies have complementary deletions so as to maintain all CREs of the ancestral gene. However, the removal of CREs leads to a sub-functionalization as CREs are critical to the spatiotemporal expression and regulation of genes.

The duplication-divergence-complementation (DDC) model explains sub-functionalization of two duplicated genes by assuming there is complementary degradation of CREs (Fig. 1). After a gene duplicates, both new gene copies start with identical CREs from the ancestral gene. Over time, these copies will relax conservation of specific CREs and those promoter regions will become selectively neutral. One of the duplicate genes will maintain the conservation of each CRE to ensure required functional regulation of this gene. Therefore, between the two duplicated copies, all original ancestral CREs are present shared between the duplicated copies. Therefore, the more rounds of duplication, the fewer shared CREs between all duplicated genes^13^. However, this is just one of the possible fates for duplicated genes.

Cytokinin Response Factors (CRFs) are a side branch of the cytokinin signaling pathway and part of the AP2/ERF TF family, which control a variety of developmental and environmental stress responses within all land plants^14,15^. All genes categorized as CRFs have a conserved CRF domain at the N-terminal and a centralized AP2/ERF DNA binding domain, as well as a CRF clade specific C-terminal region^15,16^. Phylogenetic analysis has indicated that CRFs should be placed into five evolutionarily diverged groups, or clades (I, II, III, IV, V) within the Angiosperms (Supplemental Fig. 1), each with unique functions, as outlined in Table 1 ^16,17^. The division of CRFs into five clades arose through gene duplications, from which Clade V is placed as sister to Clades I through IV. Throughout the evolution of Angiosperms, duplications within individual clades resulted in one or two genes in each of Clade I, II, III, and IV per species, and Clade V having upwards of four or more CRF genes per clade^16^. Within each species, there are additional independent CRF duplications, causing variation in number of CRFs per species^15^. A few CRF genes cannot clearly be placed into any one of the five clades, due to the lack of a C-terminal protein region. One example of this is seen in *Brassicaceae*, where there are several copies of a truncated CRF gene, (labeled as “Uncladed Brassica CRFs”).

**Table 1:** An overview of previous research conducted on Cytokinin Response Factors, including if they can be induced by cytokinin and elucidated functions for each clade with citations.

Clade	Cytokinin Induced?	Regulations and Roles
I	Yes, strongly	Salt, cold, lateral root development, cytokinin^17,18,20,28^
II	No	Cold, auxin, nitrogen, lateral root development^18,22,37^
III	Yes, strongly	Salt, oxidative stress, delayed senescence, cytokinin^15,19,21^
IV	Yes	Salt, ethylene, disease resistance, cytokinin^20,38–40^
V	Yes	Root and shoot growth (unpublished)
Uncladed Brassica CRFs	No	Root development, phosphate starvation response^41^

Brassicaceae lacks Clade IV sequences, but instead has a group of “uncladed” sequences, seen in their own row above.

General examination of CRF expression in *A. thaliana* and *Solanum lycopersicum* (*Sl*) indicated that most CRFs are expressed in several tissues throughout the plant^14,17,18^. Later experiments using transgenic promoter::GUS lines narrowed down CRF expression to primarily vascular tissue within these same tissues^19–22^. An initial cursory cis-regulatory analysis was previously conducted to elucidate possible regulatory mechanisms that control all CRF vascular expression. A highly conserved (CT)^n^ motif was found multiple times within every upstream CRF sequence, which has been linked to vascular expression when found in gene promoters^16,23^. Nearly all direct experimental examination of CRFs has been limited to Arabidopsis (AtCRFs or often simply noted as CRFs) and tomato (SlCRFs), summarized later in this work.

Research conducted in this manuscript was done to broaden our understanding of CRFs in land plants ranging from *Bryophytes* to *Brassicaceae*, by performing a detailed analysis of cis-regulatory regions for each CRF clade and provide insight into specific clade function. This *in silico* approach allowed more plant species to be examined than could easily be analyzed in the laboratory. Motif analyses of 1000 bp upstream sequences revealed novel roles and key CREs for individual CRF clades and helps in understanding the evolution of CREs in CRFs.

## Results

**Figure 2:**
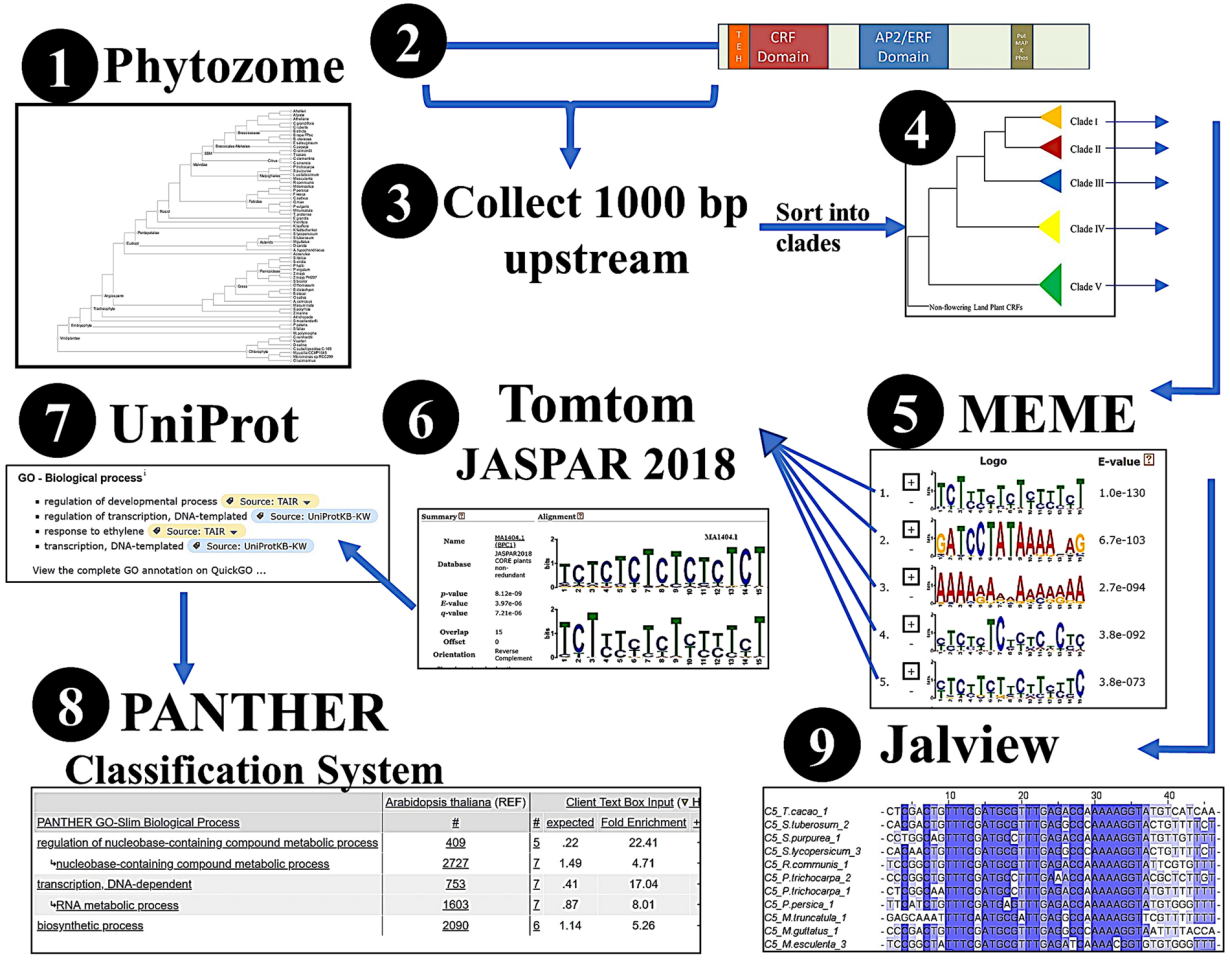
Workflow pipeline. (**1**) Cytokinin Response Factor sequences were collected from Phytozome and then checked for the conserved CRF and AP2/ERF domains. (**2**) 1000 bp upstream for each CRF sequence were collected and the amino acid sequences were compared to known A. thaliana CRF sequences (**3**) to place into their respective clade. (**4**) All upstream sequences for each clade were run through MEME. (**5**) Resulting motifs were searched through Tomtom JASPAR Core plants (**6**) to find matches to known motifs. For each motif, the biological GO terms were collected (**7**) and PANTHER GO analysis was performed. (**8**) Additionally, an alignment for each motif was created using Jalview to show percent conservation at each position. (**9**) Images in boxes at specific pipeline steps are example output from the workflow.

**Figure 3:**
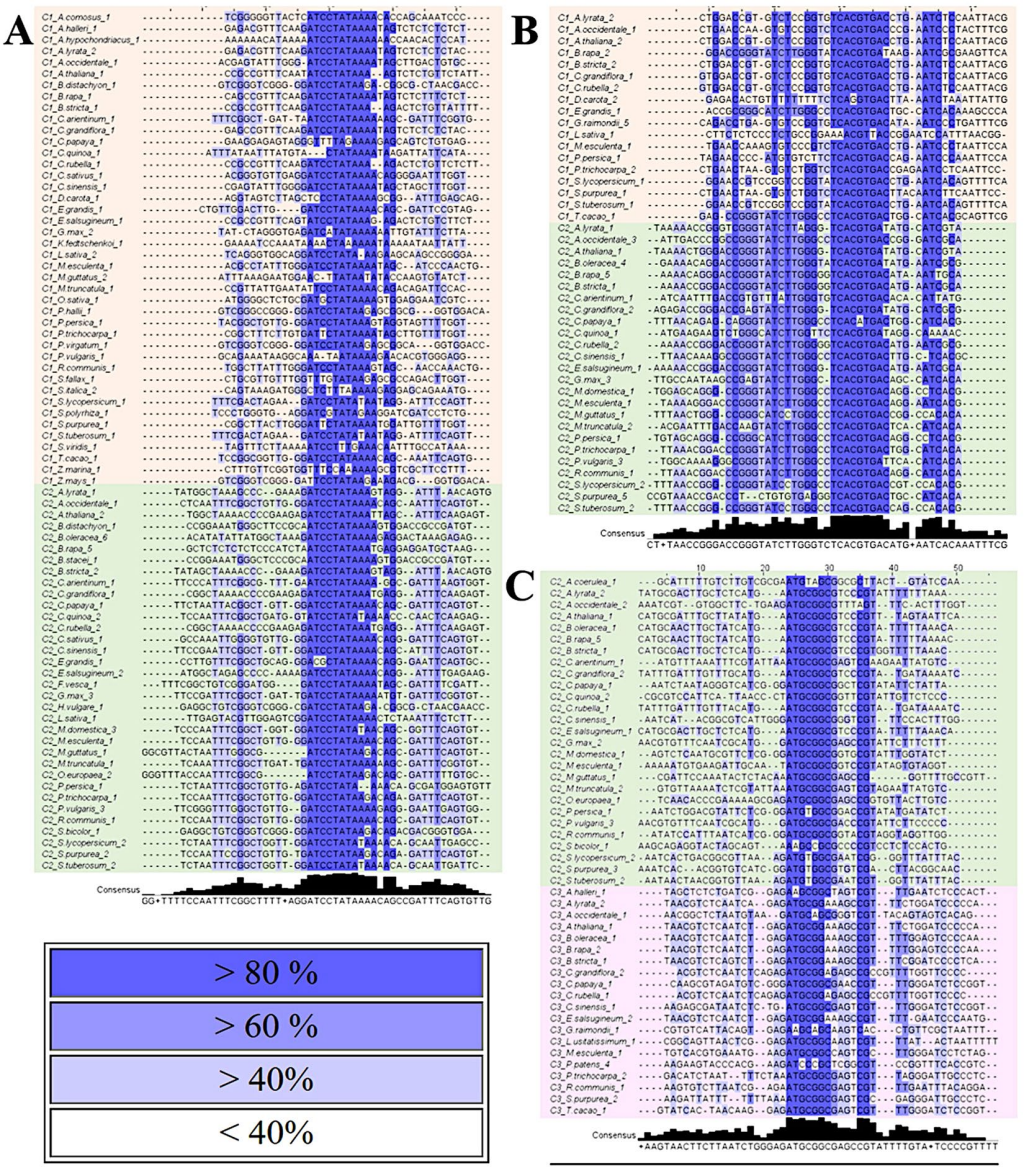
Alignment illustrating the percent conservation at each site. The darker the shade of blue, the higher the level of conservation at each site, with the most common color above representing more than 80% of sequences have the same nucleotide at that position. Both (**A**) and (**B**) are motifs seen in Clade I (orange) and Clade II (green) CRF sequences and are in the top five motifs returned by MEME. (**C**) is a motif seen in both Clade II and Clade III (pink) CRF sequences.

To conduct the motif analysis, a novel bioinformatics pipeline was created (Fig. 2). 59 plant genomes were examined revealing a total of 346 CRF sequences (Fig. 2.1–3, Table 2). For each of the five CRF clades, a MEME motif analysis identified the five most significantly enriched motifs (Fig. 2.5), which were then compared to known TF binding sites, using Tomtom: JASPAR (Fig. 2.6). To extrapolate motif functional roles, UniProt and Panther GO analysis were utilized. Separately, the sequences selected for each motif were aligned in Jalview to identify the level of conservation at each basepair position (Fig. 2.9). E-values of the identified statistically significant motifs ranged from 9.1 × 10^−34^ to 1.6 × 10^−280^, while random shuffled sequences all had non-significant E-values above 8.4 × 10^6^.

**Table 2:** Plants used to isolate CRF sequences and their respective upstream sequences for this study from Phytozome.

Amaranthus hypochondriacus	Citrus sinensis	Panicum hallii
Amborella trichopod	Cucumis sativus	Panicum virgatum
Anacardium occidentale	Daucus carota	Phaseolus vulgaris
Ananas comosus	Eucalyptus grandis	Physcomitrella patens
Aquilegia coerulea	Eutrema salsugineum	Populus trichocarpa
Arabidopsis halleri	Fragaria vesca	Prunus persica
Arabidopsis lyrata	Glycine max	Ricinus communis
Arabidopsis thaliana	Gossypium raimondii	Salix purpurea
Asparagus officinalis	Hordeum vulgare	Setaria italica
Boechera stricta	Kalanchoe fedtschenkoi	Setaria viridis
Brachypodium distachyon	Kalanchoe laxiflora	Solanum lycopersicum
Brachypodium stacei	Linum usitatissimum	Solanum tuberosum
Brassica oleracea capitata	Malus domestica	Sorgum bicolor
Brassica rapa	Manihot esculenta	Sphagnum fallax
Capsella grandiflora	Marchantia polymorpha	Spirodela polyrhiza
Capsella rubella	Medicago truncatula	Theobroma cacao
Carica papaya	Mimulus guttatus	Trifolium pratense
Chenopodium quinoa	Olea europaea	Zea mays
Cicer arientinum	Oryza sativa	Zostera marina
Citrus clementina		

### Conservation of motifs within each clade

Two types of MEME analyses were used to elucidate motifs: those found in every upstream sequence in a given data set or OOPS and those found within most, but not all, upstream sequences or ZOOPS. One motif, a (CT)^n^ or (GA)^n^, was repeatedly found in all clades when analyzing CRF clades individually, except for the uncladed *Brassicaceae* sequences. The examination of Clade I found that four of the top five motifs were either (CT)^n^ or (GA)^n^ for OOPS and ZOOPS MEME analyses. The remaining top motif for Clade I is similar between OOPS and ZOOPS showing a conserved pattern of “GATCCTATAAA” and a noticeable lack of conservation flanking either side. The remaining Clade I OOPS motif has stronger conservation across the 25 bp motif, with the main motif pattern of “TCACGTGAC”.

In the examination of Clade II OOPS MEME results, only the second highest of the top five motifs returned was (CT)^n^ or (GA)^n^. The top motif is seen in both Clade II OOPS and ZOOPS, with a strong nucleotide pattern of “GATCCTATAA” followed by a degradation of nucleotide conservation. The top motif for Clade II ZOOPS has conservation seen throughout the 25 bp motif, with eight nucleotides strongly conserved with no variability, “TCACGTGA” and was found in 44 or 85 Clade II sequences examined. Clade II ZOOPS had two other motifs with noticeable conservation, one featuring “ATGYGGCG” with significant degradation of conservation flanking either side, and the other motif having much stronger conservation and a conserved core of “CTGANTCAGCA”.

Much like in CRF Clade I and II, Clade III OOPS and ZOOPS analyses identified (CT)^n^ or (GA)^n^ motifs in the top five motifs. The top motif found in Clade III upstream CRF sequences has weak conservation followed by a relatively conserved core of “RARAWGCGGMNAGYCGYY” with a strong E-value. A less conserved, but still nearly identical, version of the motif is seen in the OOPS analysis, meaning that all Clade III CRF upstream sequences have this motif. The next ranked motifs for Clade III OOPS and ZOOPS were also identical, “TTNCTTGG” followed by several non-conserved nucleotides and “RYCAAG” at the other end. The most conserved motif seen in Clade III upstream sequences, “CNTTTTGACTCTTC”, was ranked fifth and is seen in 65% of Clade III sequences. The fifth ranked motif seen in all Clade III sequences was a run of 16 A’s with lower conservation at 3 positions.

Clade V motifs had the strongest E-values among clades including the highly conserved (CT)^n^ or (GA)^n^ motif seen in all clades. Interestingly, the motifs called for both OOPS and ZOOPS were identical.

When looking exclusively at the upstream sequences of the *Brassicaceae*, uncladed CRFs had motifs with E-values that were overall weaker than Clades I-V. The motifs and patterns within *Brassicaceae* are rather unique compared to other clades and are discussed in more detail in subsequent sections.

### Conservation of motifs shared between CRF clades

Examinations of all plant samples revealed three instances where identical motifs were independently identified in two different clades, indicating they have been conserved from duplication events from which CRFs in distinct clades arose (Fig. 3). Two of the motifs were seen in every Clades I and II sequence. This is shown in more detail in Fig. 3A for the second most statistically significant motif for both Clade I and Clade II results. Additionally, Clade II sequences have a higher level of conservation flanking either side of the motif (Fig. 3A,B). Furthermore almost all sequences feature a “GC” at position 30 and 31, except for the Clade II *Brassicaceae* sequences (Fig. 3C), which are uniquely derived with many distinct motifs compared to the rest of the plant taxa within an individual clade noted below.

### Distinct motif conservation within *Brassicaceae*

**Figure 4:**
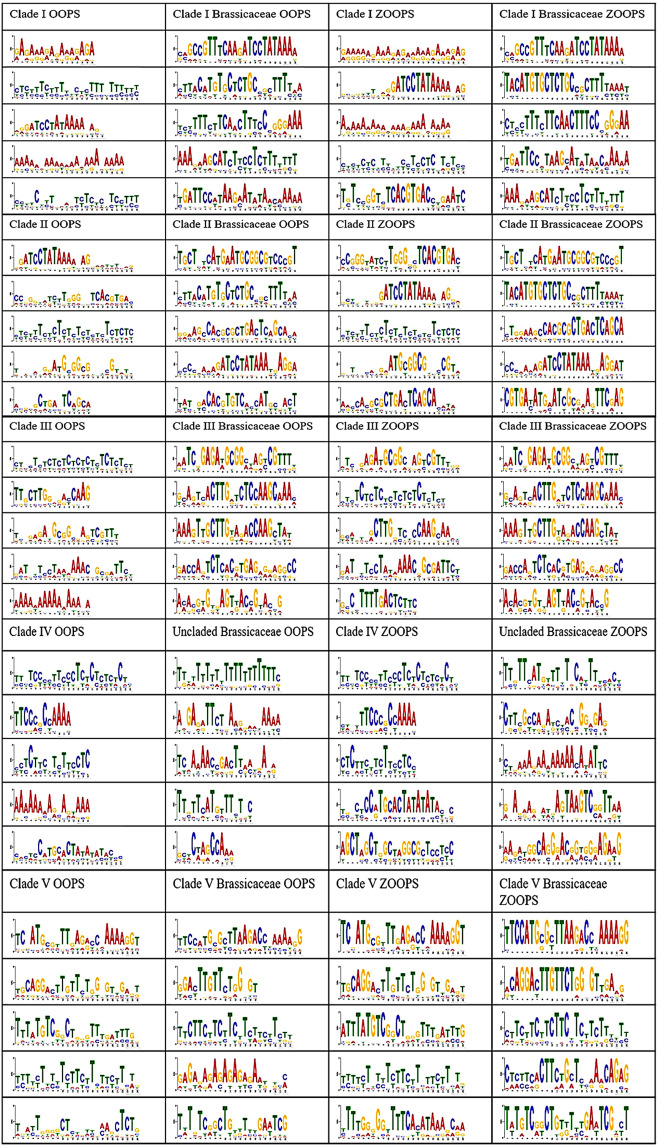
Comparison of motifs between all plants and only Brassicaceae, where every CRF sequence given must have every motif MEME finds (OOPS) and allowing for some sequences not to have every motif found (ZOOPS). The level of conservation varies drastically between the two different groups, as noted by the decreased frequency where a position can wobble between nucleotides at that given site.

A majority of work conducted on CRFs has been on *A. thaliana*, creating difficulties when extrapolating results onto other plants. In order to analyze the differences in cis-regulatory regions between all plant taxa and just *Brassicaceae*, to which *A. thaliana* is a member, *Brassicaceae* sequences alone were analyzed using the pipeline (Fig. 2). While the conservation of CREs seen within and between clades for all land plants is significant, the conservation of CREs seen exclusively in *Brassicaceae* species is considerably greater. Motifs found from evaluations forcing each plant species to have every motif present (OOPS), typically results in having a few nucleotides of strong conservation surrounded by regions of large nucleotide variation (Fig. 4). The Clade I OOPS motifs for all plant species MEME analysis were primarily TCTC repeats. However, when just *Brassicaceae* were analyzed, only one of the top five motifs was a TCTC repeat, indicating more complex patterns are being conserved. CREs found using exclusively *Brassicaceae* upstream sequences are characterized by stronger nucleotide conservation at each site with significantly less wobble or nucleotide variation seen compared to all plants. Clades I, II, III and V CRF upstream sequences all have regions of considerable nucleotide conservation (Fig. 4). Additionally, the identified motifs illustrate the differences between Clade IV and the uncladed *Brassicaceae* CRFs.

### Functional roles of identified conserved CRF motifs

**Figure 5:**
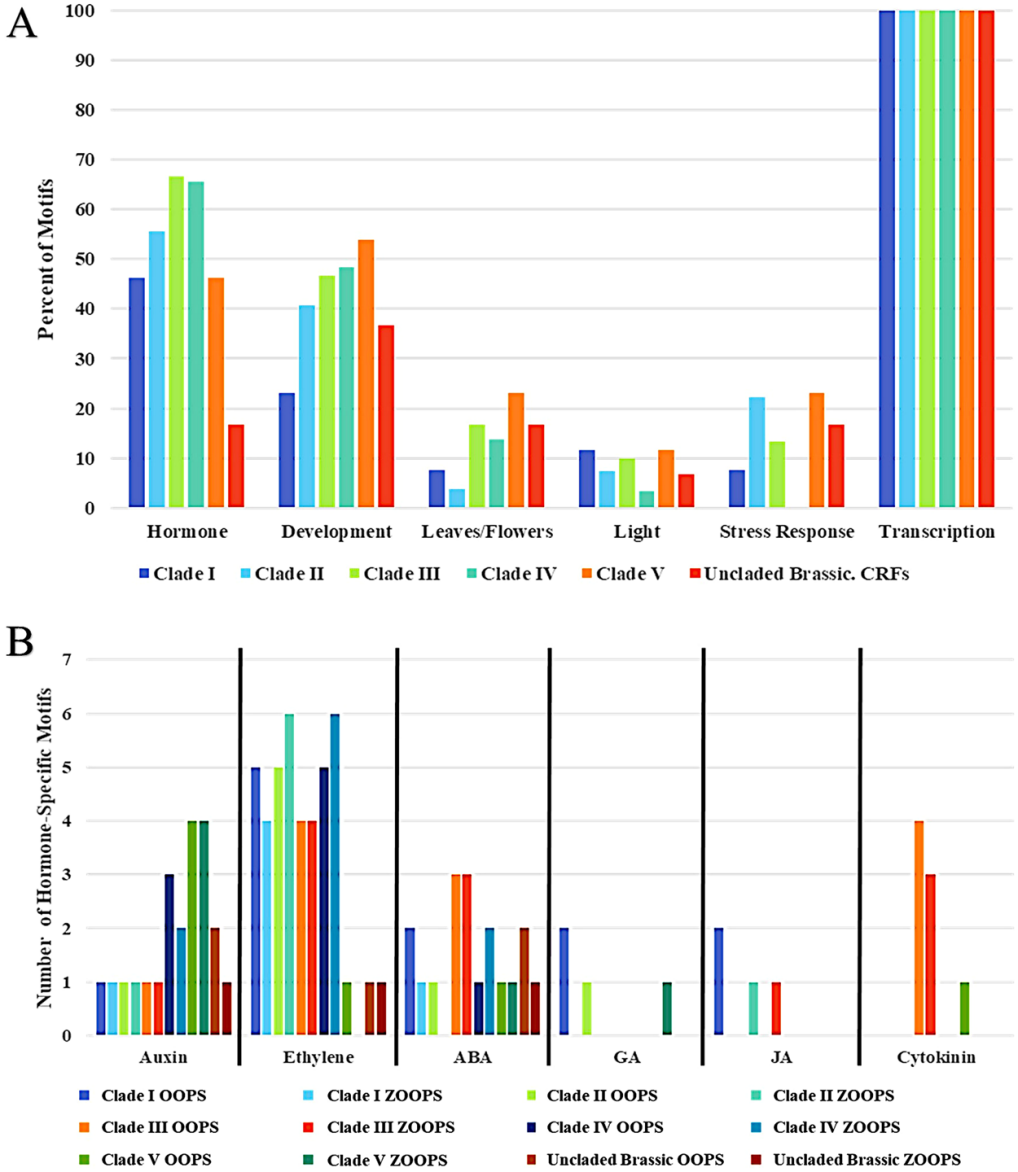
(**A**) The six main functional categories for each ZOOPS motif matched to, based upon Tomtom, Uniprot, and Panther GO analysis, based upon frequency in the top 15 Tomtom results for each Clade sequences. Motifs could be classified into more than one category, as transcription factors often have many roles and the top three matches for each motif were examined. (**B**) A breakdown of which hormones are seen most commonly within each clade and overall for both OOPS and ZOOPS.

Individual clade motifs were further analyzed by utilizing Tomtom, Uniprot, and Panther DB to compare to known TF binding sites and any previously determined experimentally function (Table 2, Supplemental Table 1). TF binding sites general could be placed into six functionally-based categories: hormone, development, flowering/leaf senescence, light, transcription regulation, or stress response (Fig. 5). The most common functional category for all motifs was hormone-related function (Fig. 5). Every motif examined was found as linked to the regulation of transcription, however, that is expected given they are transcription factor binding sites.

Additional manual in-depth analysis of Clade motifs indicates that expression of Clade I CRF sequences should be responsive to ethylene, abscisic acid, and brassinosteroid hormones. Motifs in this clade were found with connections to root and overall plant development, along with flowering time and stomatal movement. Of the fifteen motifs analyzed for Clade I, 46% were related to hormones and 23% to development (Fig. 5). Clade II similar to Clade I, has 55% of motifs relating to hormones (Fig. 5). Unique to Clades II and III is a motif connected to regulation of root growth and stomatal movement. The only exclusive Clade II motif functions in RNA splicing and processing, DNA repair, regulation of cell cycle and differentiation, along with response to auxin, bacteria, fungus, and nematodes. Nearly a quarter of all motifs (22%) for Clade II aid with stress response, the highest among clades (Fig. 5). Clade III motifs are connected to several hormones: cytokinin, ethylene, auxin, and abscisic acid. Additionally, Clade III motifs show a major linkage (46%) to influencing development (Fig. 5). Clade IV motifs had no motifs found as related to light or stress response. Of all Clade IV motifs, 66% were hormone-related and 40% influenced development (Fig. 5). Ethylene was the primary (75%) hormone motif, with abscisic acid and auxin composing the remaining 25%. Clade V motifs were uniquely connected to the hormone gibberellic acid (GA), as well as having their largest connection to development, making up the top percentage of categories at 53% (Fig. 5). Leaves/flowering related motifs are present in 42% of Clade V results, which is the highest percentage for leaves/flowering compared to Clades I-IV (Fig. 5). Clade V tied with Clade I for lowest percentage of hormone-related motifs among Clades I-V, with only 46%. Motifs seen in Clade V are non-overlapping with other clades, except for the top motif seen in all CRF sequences, which is expected given it is sister to the other CRF clades.

When all CRF upstream sequences were examined together, 54% of motifs related to hormone regulation, slightly below the overall average of 58%. Development was seen in 27% of motifs, while flowering/leaf senescence, light, and stress response were all only seen in 9% of potential TF roles. Ethylene is overwhelmingly the most commonly seen hormone regulated across all Clades, with cytokinin seen once, and no other plant hormones seen in resulting TF binding site matches (Fig. 5).

### Evolutionary divergence of duplicated CRF upstream sequences

**Figure 6:**
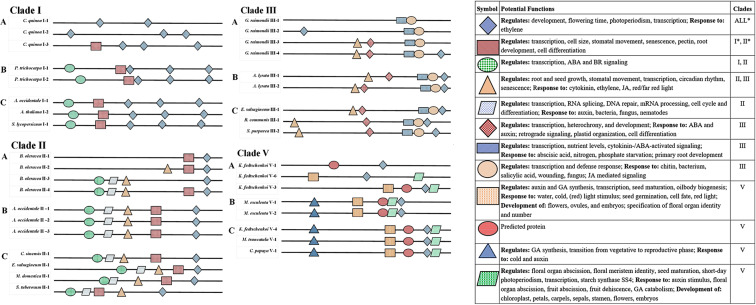
Pattern of conserved motifs (A) panels illustrate evolutionary divergence in duplicated CRF sequences. (**B**) sequences are duplicated CRF sequences where both copies have retained matching motifs. (**C**) A collection of different plant species, all with matching copies of motifs found for their respective clade. (Right) A summary of functions found for each motif, represented by a symbol. Functions were broken down into “regulates”, “response to”, and “development of” categories. An *indicates that motif is present in both OOPS and ZOOPS MEME runs, meaning all sequences in that respective clade has that motif.

In order to examine the potential change in CRE patterns, duplicated CRF genes from individual species were identified and compared. While this type of examination is regularly conducted on motifs or domains of protein sequences, it is rarely done for promoter sequences possibly because promoter sequences and intragenic are thought of as less conserved as exon coding sequences. Despite this, our analysis of 346 CRF sequences from 59 plant species revealed an abundant conservation and divergence of CREs, which fall into four main patterns of duplication events. In two of the duplication event patterns, CRF genes have the same top five upstream motifs present: one in the exact same order, with the other different order (Fig. 6B,C). In the third identified pattern, each duplicated promoter sequence has a unique motif not seen in the other copy, while remaining motifs are identical. This pattern could indicate a sub- or neo-functionalization between duplicated CRF genes, as TF binding sites in the upstream sequence play large roles in gene identity. In the fourth identified duplication event pattern, promoter sequences have serial or complementary deletions, similar to the DDC model that together make up what is likely the “original” motif pattern (Fig. 6A). From these patterns, those having identical sequence motifs (Fig. 6B) or serial deletions of motifs (Fig. 6A) are the most commonly found. In plant species that had more than the average number of CRF sequences per clade, more than one pattern was commonly identified, indicating there is not necessarily a single evolutionary model at work in cis-regulatory regions. However, regardless of pattern, the statistical significance of each motif and the level of pattern conservation between plant species indicates CREs can and should be examined for duplication and divergence more broadly.

## Discussion

### Cis-regulatory elements are evolutionarily conserved in Cytokinin Response Factors

Prior to this study, limited research was conducted analyzing the evolutionary conservation of CREs within a highly duplicated gene family. Although extensively conducted on protein sequences, the prevailing wisdom that cis-regulatory regions are not highly conserved combined with the lack of fully sequenced genomes seems to have limited interest in conducting such efforts. However, this study shows that CRE motif analyses should be conducted in a widespread manner, as it provides valuable information regarding regulation of genes. Furthermore, the importance of motifs found using this research pipeline provides novel avenues for investigation of CRF gene function (Fig. 2). Finally, the level of conservation identified from examination of duplication and divergence of CREs indicates the need for broader research focused on cis-regulatory regions of duplicated genes from many different gene families.

Duplications of CRF genes has spanned across evolutionary time originating with an ancestral CRF found in species after the emergence of plants on land and becoming widespread within Angiosperms. This expansion has also occurred through individual gene duplications within a species, which lend cis-regulatory regions to higher levels of mutation and divergence, since intragenic regions are not as highly conserved as genetic coding sequences. The identification of five evolutionary preserved motifs of up to 25 bp shows a level of promoter sequence conservation in CRFs similar to that found in coding regions.

Even more potentially noteworthy are the motifs which have been conserved between Clades I and II and Clades II and III (Fig. 3). These duplications have occurred in the evolutionary timeframe from the origin of Angiosperms to present day and illustrate the level of conservation seen within the CREs found in this study. Analyzing these differing motifs present within each of these clade pairings, the divergence of CREs between two clades after their duplication can be elucidated. This pipeline created for this study of CRFs could easily be adapted and applied to the upstream and downstream cis-regulatory regions of other groups of conserved duplicated genes to analyze CREs across an evolutionary landscape.

### Differing motifs within all plants compared to the *Brassicaceae* alone

While the conservation of CREs across all land plants is significant considering evolutionary forces, an examination specifically focused on the *Brassicaceae* revealed an even stronger level of conservation present in CREs within this group. An interesting dichotomy arises due to the high level of conservation seen in the *Brassicaceae*, which is not present when comparing the Brassica-specific elements to those in other plant families. As such, this is potentially problematic when trying to extrapolate research results from well-studied members of the *Brassicaceae*, specifically *A. thaliana*, to plants from other families.

In fact, interesting differences were found when examining functional roles attributed to specific CRF clades from solely *Brassicaceae* sequences versus all plant species (Fig. 5 and Supplemental Fig. 2). This could be specifically seen within functional attributed roles to distinct hormones. When attributing a functional role to a clade for the plant hormone ethylene from analysis of all plant sequences (Fig. 5), this was found for Clades II and IV sequences but not in Clade V sequences. However, a similar examination using just *Brassicaceae* sequences identified ethylene as most common attribute in Clade V sequences. Likewise, for the hormone ABA, Clade II sequences lacked such motifs, yet in *Brassicaceae* only sequence analysis, Clade II had the highest number of motifs relating to ABA. However, these results were based on presence or absence of motifs in the five most highly ranked sequences, so a deeper analysis could still show such conservation. While such differences may be expected due to evolutionary selection forces unique to the *Brassicaceae* among plants, this difference suggests the need for researchers to be mindful of experimental studies focused solely on one plant system.

### Hormone-related motif roles

Previous experimental research has shown CRF Clades I, III, IV, and V genes are transcriptionally regulated by cytokinin and identification of cytokinin-related CREs here supports these results^14,17,19,21^. Within the top five motifs found, both Clade III and Clade V sequences have motifs that indicate cytokinin-related TFs can bind. Although not in the five motifs for Clade I, there was a motif in the top ten motifs with similarity to the canonical GCC box, falling into the AP2/ERF TF family, which and is known to aid with the response to cytokinin (Supplemental Fig. 3)^14,24,25^. Not every motif found by MEME is necessarily a TF binding site and motifs not within the top five MEME results are still worth looking at for matching motifs to connect to previously conducted experimental results (Supplemental Figs 3–7).

Other plant hormones, such as auxin, ethylene, abscisic acid (ABA), gibberellic acid (GA), and jasmonic acid (JA), support a pattern of intricate and complicated regulation. CRFs are a side branch of the cytokinin signaling pathway and it is expected they would share an equally complex interaction with the other plant hormones. Only auxin-related elements were found in MEME analysis, both OOPS and ZOOPS, in every Clade and the uncladed *Brassicaceae* CRF sequences. Auxin and cytokinin are antitheses, working in conjunction to regulate many plant processes. Specifically for CRFs, research has been conducted linking CRFs with auxin transporters^26^.

Other hormones-related motifs were found in all clades except for Clade V ZOOPS and Clade II ZOOPS, lacking ethylene and ABA motifs respectively. Importantly, by design ZOOPS does not require all upstream sequences to have each motif, while OOPS does. Therefore, Clade V ZOOPS and Clade II ZOOPS MEME analysis lacking ethylene and ABA-related motifs, respectively, is not significant as Clade V OOPS and Clade II OOPS both having these motifs. Experiment based analysis are still required to fully verify these predicted functional roles for CRFs.

### Development-related motif roles

Development was the second most frequent identified motif category, which is an often-attributed functional role for AP2/ERF TFs^27^. Several recent studies have analyzed the phenotypic effects of both CRF overexpressors and mutants. *At*CRF1, *At*CRF2, *At*CRF3, *At*CRF5, and *At*CRF6 have all been linked to primary and lateral root and shoot growth, embryo development, leaf senescence, hypocotyl elongation, and rosette size^21,26,28,29^, indicating that proper spatiotemporal expression of CRF genes are critical for normal plant development. The TFs that bind to the upstream regions of the CRF sequences analyzed in this study play a key role in maintaining spatiotemporal expression of each CRF, and therefore, helping to regulate normal development.

### Conclusions and future directions

Through the creation of this novel pipeline, promoter regions can be analyzed for CREs to indicate potential functions of a gene and reveal conservation or divergence of CREs. Over 300 promoter regions for CRF genes were collected and analyzed with MEME Suite to find numerous conserved CREs. The motifs for each individual CRF clade showed strong patterns of conservation, suggesting sub-, or neo-functionalization. Many of the previously conducted experiments on CRFs support the motifs that were elucidated though this novel pipeline, bolstering their significance. The conservation of motifs within each CRF clade, even when using wide-ranging Angiosperms species, is considerable, especially given its long been thought that cis-regulatory regions are not conserved in a manner similar to coding regions. However, validation of what TFs are binding to each motif should still be conducted using a combination of both serial deletion of promoter regions and bioinformatic techniques to capture TFs binding to cis-regulatory regions. By using a variety of Angiosperms, the evolutionary changes of cis-regulatory regions can continue to be understood and later applied broadly to other plant families. Outside of the CRF TF family, this innovative pipeline can be applied to upstream, downstream, or intron regions of gene for families within plants or families outside of plants.

## Methods

### Collection of sequences and analysis for presence of common motifs

Cytokinin Response Factors (CRF) sequences were identified via tBLASTn on Phytozome (https://phytozome.jgi.doe.gov) (Supplemental Fig. 8) using known *A. thaliana* CRF sequences (Fig. 2). Sequences were verified as CRFs by checking for the presence of previously identified conserved CRF domain^15^ and AP2/ERF domain within the coding sequence^27,30^, after which 1000 bp upstream of the transcriptional start site plus any 5’ untranslated regions and the entire exon sequence were collected (Fig. 2.2,3). The collected exon and upstream sequences were sorted into the five distinct groupings (“clades”) by searching the exon sequence on NCBI BLAST and comparing to known CRF genes (Fig. 2.4). Varying subsets, including clade and plant family groups, were analyzed using MEME suite tools^31^ to identify common motifs in these sequences. For each subset of upstream sequences, motifs appearing once in all sequences (OOPS) or in most, but not all, sequences (ZOOPS) were collected. Motifs were allowed to be from 5 to 25 bp in length, with an E-value less than 0.05, the default parameter for MEME (Fig. 2.5). For validation, all sequences collected were shuffled and run through MEME using identical parameters, which kept the same sequence composition as the upstream sequences analyzed in this study.

### Functional categorization of identified common motifs

Collected motifs were run through TomTom^32^, a part of MEME Suite, via the JASPAR Core Plants (2018) database^33^ using default parameters (Fig. 2.6). For the top three results from TomTom, Uniprot IDs were collected, with a p-value significance cut off value of 0.01 (Fig. 2.7). These Uniprot IDs were then used to collect biological GO terms for analysis of potential functions for each motif and for analysis of each of the CRF groups. PANTHER^34^ was used to analyze GO terms for statistically significant overrepresentation for each clade using default parameters (Fig. 2.8).

### Motif conservation alignment

For every plant containing a specific significant motif, the 5 to 25 base pair motif plus 10 bp on either side were loaded into Jalview^35^. Sequences were then aligned using Clustal^36^, within Jalview, using default parameters. Color settings were changed to highlight based on percent identity, which illustrates level of conservation based upon sequences provided for each alignment (Fig. 2.9).

## Supplementary information

Supplemental Information

## Data Availability

All data generated or analysed during this study are included in this published article (and its Supplementary Information Files).
