# Supplementary material for: Lineage specific conservation of cis-regulatory elements in Cytokinin Response Factors
Source: Sci Rep. 2019 Sep 16;9:13387. doi: 10.1038/s41598-019-49741-6 (PMC6746799; doi:10.1038/s41598-019-49741-6)
Supplement: Supplementary file 1 — Supplemental Information [file 41598_2019_49741_MOESM1_ESM.pdf]

Supplemental Information for

Lineage specific conservation of cis-regulatory elements in Cytokinin Response Factors

By Rachel V. Powell, Cipher R. Willett, Leslie R. Goertzen, Aaron M. Rashotte

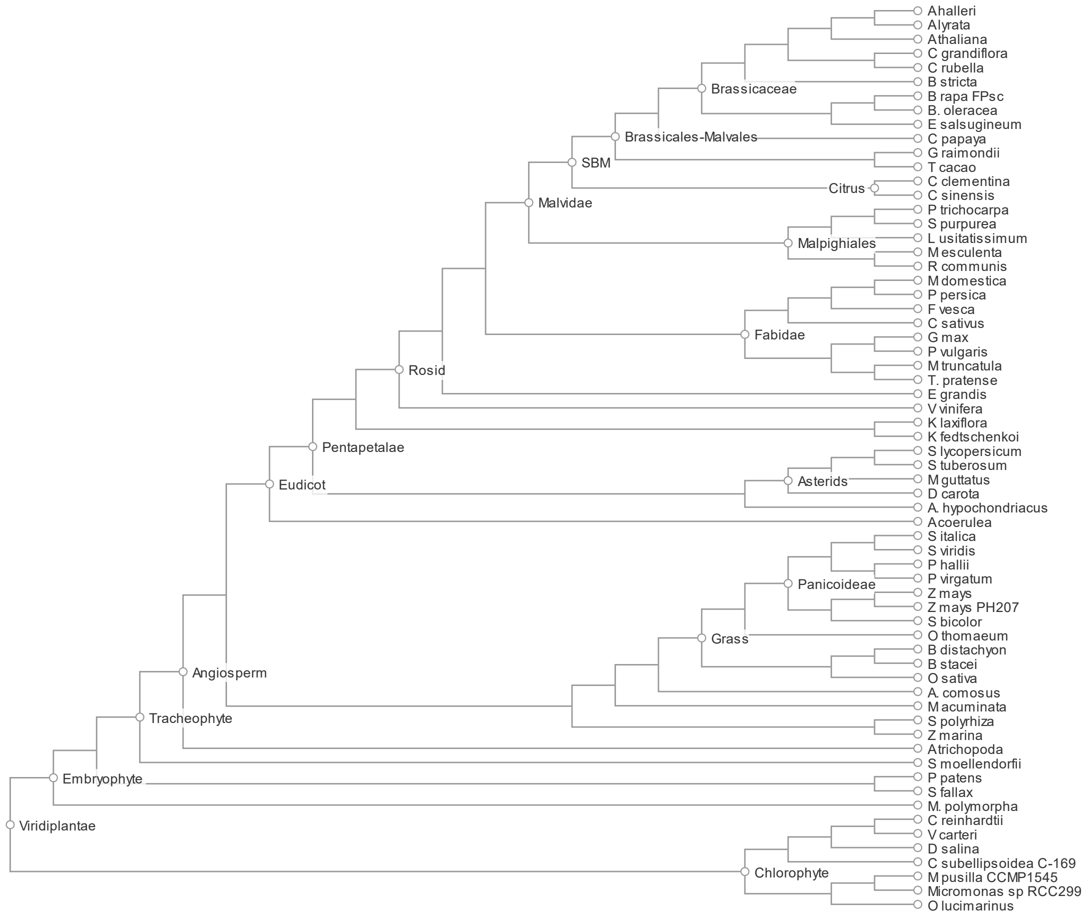

Supplemental Figure 1: A phylogeny of plant genomes available from Phytozome used for examination in this study

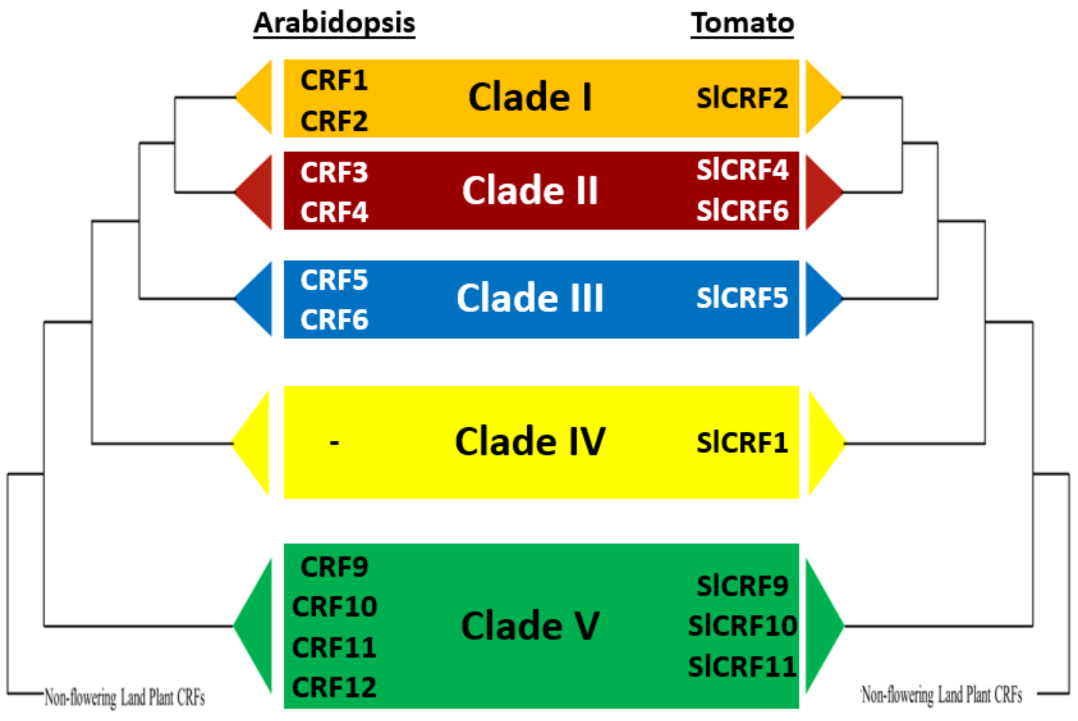

Supplemental Figure 2: A simplified diagram indicating Arabidopsis and tomato CRF gene orthologs as sorted by clade

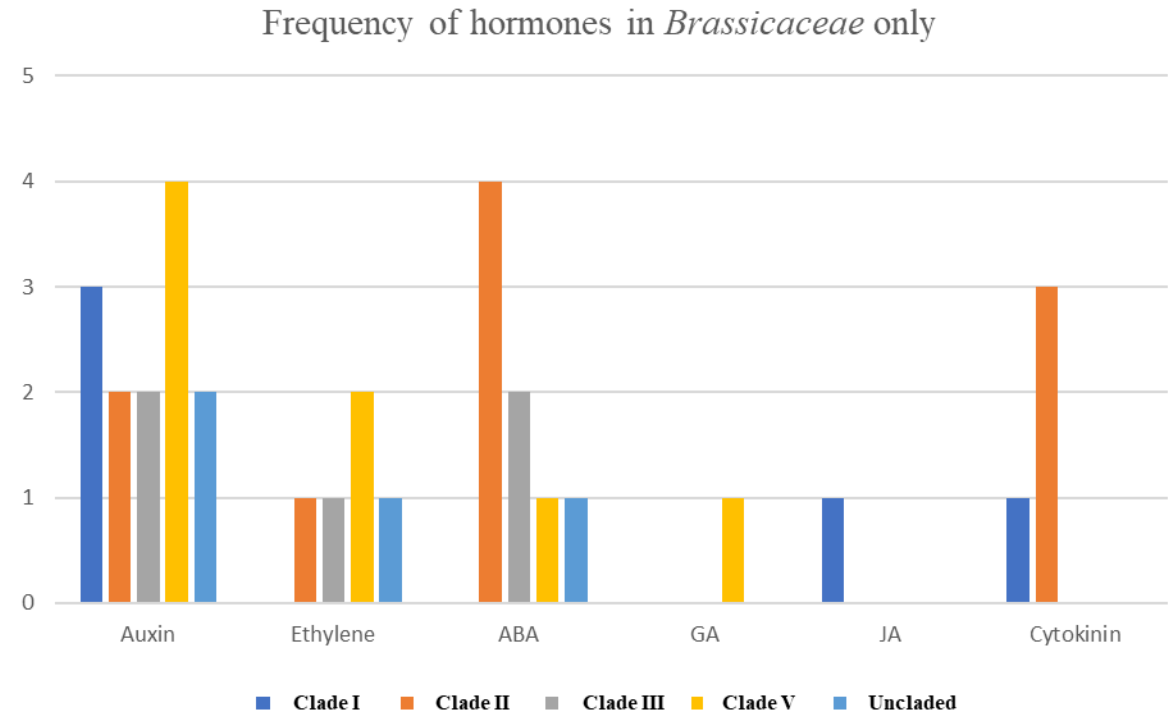

Supplemental Figure 3: A breakdown of hormone-related cis-element frequencies identified in Tomtom analyses only within the Brassicaceae genomes examined.

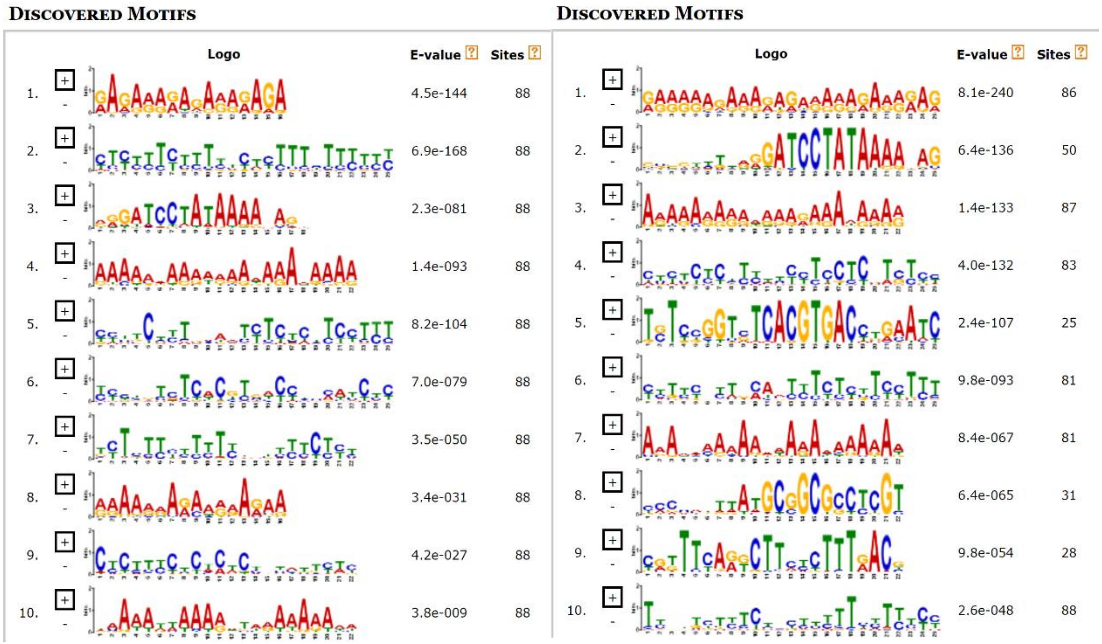

Supplemental Figure 4: Clade I top 10 motifs identified in Tomtom analysis from all genomes using OOPS (left) and ZOOPS (right)

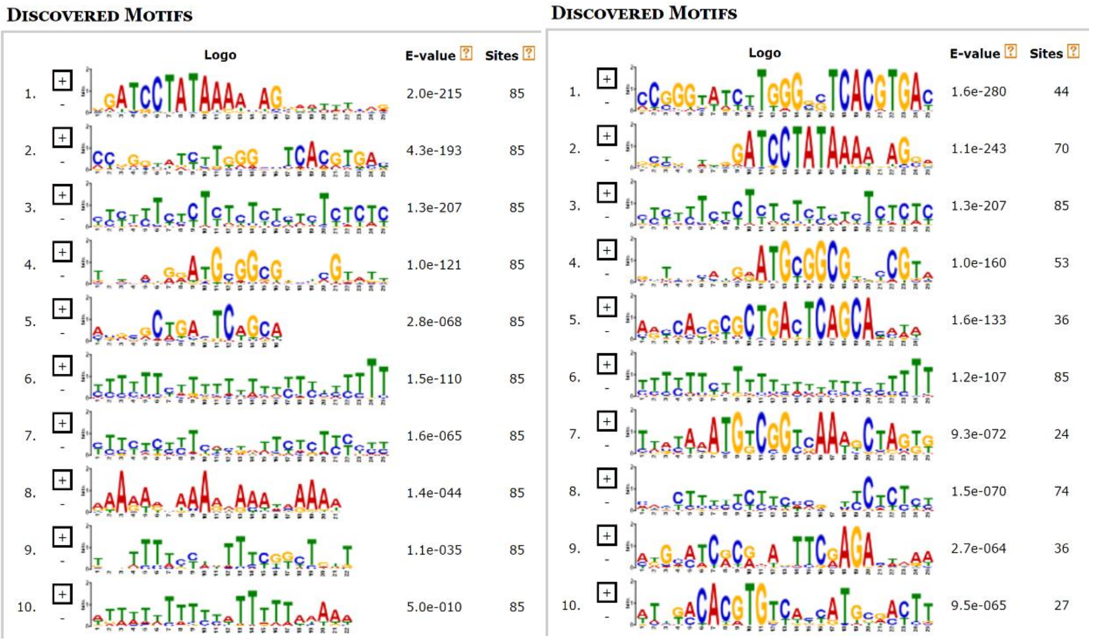

Supplemental Figure 5: Clade II top 10 motifs identified in Tomtom analysis from all genomes using OOPS (left) and ZOOPS (right)

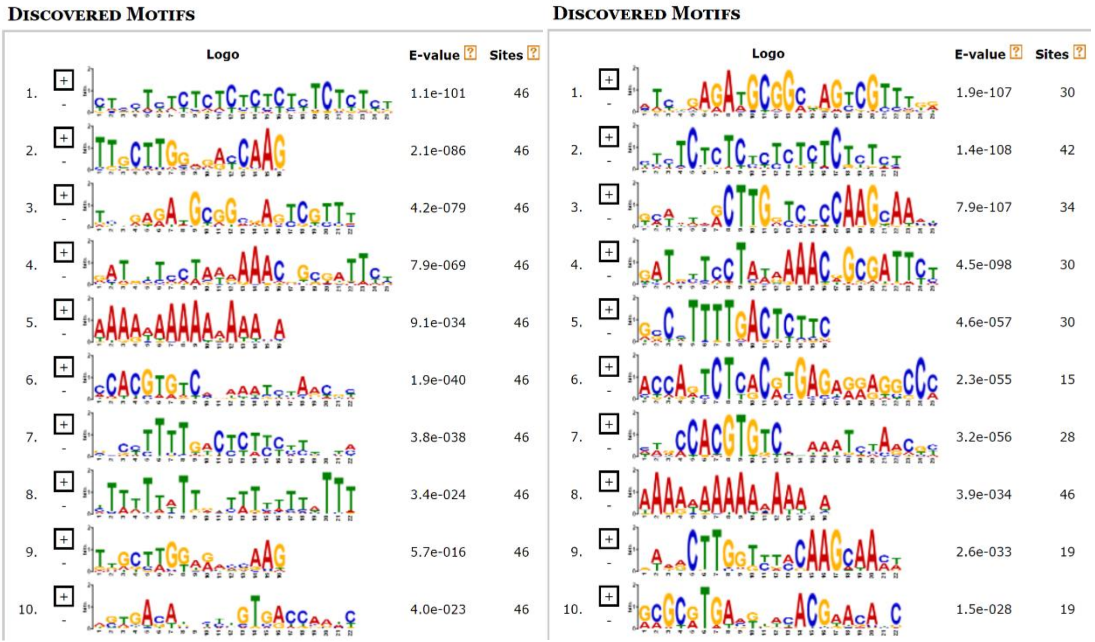

Supplemental Figure 6: Clade III top 10 motifs identified in Tomtom analysis from all genomes using OOPS (left) and ZOOPS (right)

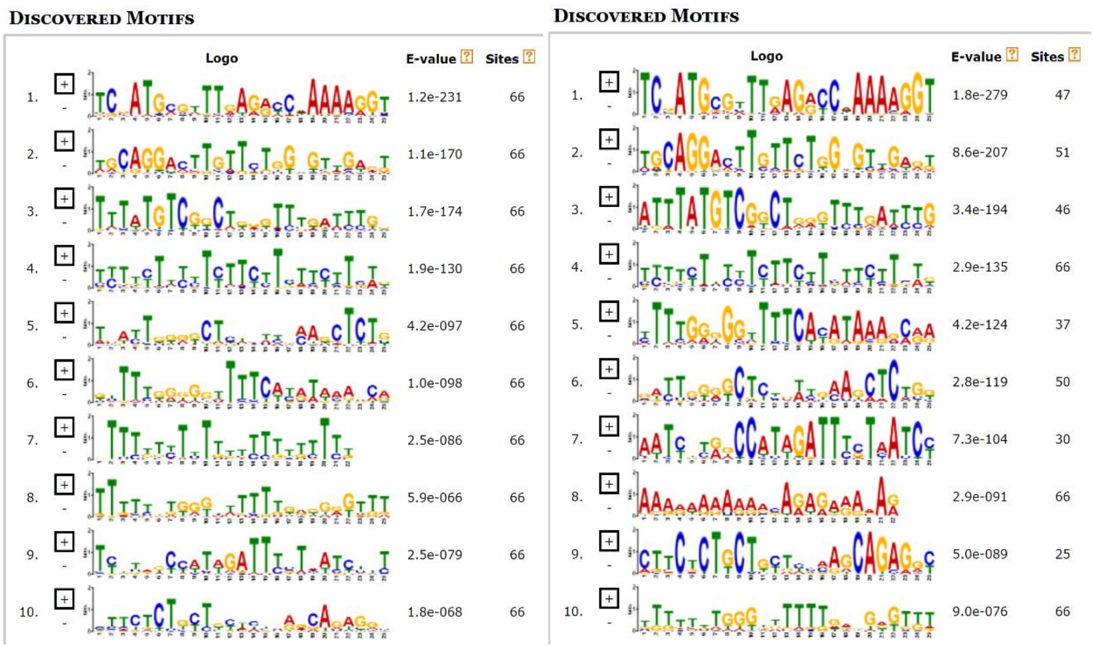

Supplemental Figure 7: Clade V top 10 motifs identified in Tomtom analysis from all genomes using OOPS (left) and ZOOPS (right)

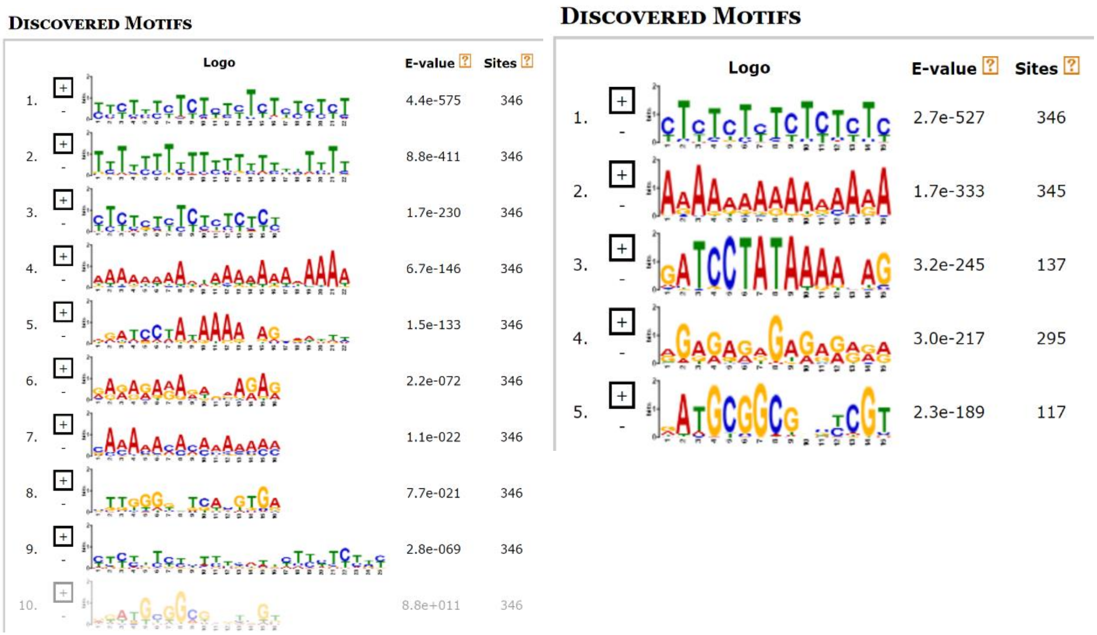

Supplemental Figure 8: All CRF upstream sequences motifs identified in Tomtom analysis from all genomes using OOPS (left) and ZOOPS (right)

Supplemental Information 1: Arabidopsis Cytokinin Response Factors

>C1_AT_AT4G11140_AA // AtCRF1

METEKKVSLPRILRISVTDPYATDSSSDEEEEVDFDALSTKRRRVKKYVKEVVLDSVVSD
KEKPMKKKRKKRVVTVPVVVTTATRKFRGVRQRPWGKWAAEIRDPSRRVRVWLGTF
DTAEEAAIVYDNAAIQLRGPNAELNFPPPPVTENVEEASTEVKGVSDFIIGGGECLRSPVS
VLESPFSGESTAVKEEFVGVSTAEIVVKKEPSFNGSDFSAPLFSDDDVFGFSTSMSESFGG
DLFGDNLFADMSFGSGFGFGSGSGFSSWHVEDHFQDIGDLFGSDPVLTV*

>C1_AT_AT4G23750_AA // AtCRF2

MEAEKKMVLPRIKFTEHKTNTTTIVSELTNTHQTRILRISVTDPDATDSSSDDEEEEHQRF
VSKRRRVKKFVNEVYLDSGAVVTGSCGQMESKKRQKRAVKSESTVSPVVSATTTTTGE
KKFRGVRQRPWGKWAAEIRDPLKRVRLWLGTYNTAEEAAMVYDNAAIQLRGPDALTN
FSVTPTTATEKKAPPPSPVKKKKKKNNKSKKSVTASSSISRSSSNDCLCSPVSVLRSPFAV
DEFSGISSSPVAAVVVKEEPSMTTVSETFSDFSAPLFSDDDVFDFRSSVVPDYLGGDLFGE
DLFTADMCTDMNFGFDFGSGLSSWHMEDHFQDIGDLFGSDPLLAV*

>C2_AT_AT4G27950_AA // AtCRF3

MMMDEFMDLRPVKYTEHKTVIRKYTKKSSMERKTSVRDSARLVRVSMTDRDATDSSS
DEEEFLFPRRRVKRLINEIRVEPSSSSTGDVSASPTKDRKRINVDSTVQKPSVSGQNQKKY
RGVRQRPWGKWAAEIRDPEQRRRIWLGTFATAEEAAIVYDNAAIKLRGPDALTNFTVQ
PEPEPVQEQEQEPESNMSVSISESMDDSQHLSSPTSVLNYQTYVSEEPIDSLIKPVKQEFLE
PEQEPISWHLGEGNTNTNDDSFPLDITFLDNYFNESLPDISIFDQPMSPIQPTENDFFNDLM
LFDSNAEEYYSSEIKEIGSSFNDLDDSLISDLLLV*

>C2_AT_AT5G53290_AA // AtCRF4

MDEYIDFRPLKYTEHKTSMTKYTKKSSEKLSGGKSLKKVSICYTDPDATDSSSDEDEEDF
LFPRRRVKRFVNEITVEPSCNNVVTGVSMKDRKRLSSSSDETQSPASSRQRPNNKVSVSG
QIKKFRGVRQRPWGKWAAEIRDPEQRRRIWLGTFETAEEAAVVYDNAAIRLRGPDALT
NFSIPPQEEEEEEEPEPVIEEKPVIMTTPTPTTSSSESTEEDLQHLSSPTSVLNHRSEEIQQV
QQPFKSAKPEPGVSNAPWWHTGFNTGLGESDDSFPLDTPFLDNYFNESPPEMSIFDQPM
DQIFCENDDIFNDMLFLGGETMNIEDELTSSSIKDMGSTFSDFDDSLISDLLVA*

>C3_AT_AT2G46310_AA // AtCRF5

MKSRVRKSKYTVHRKITSTPFDGFPKIVKIIVTDPCATDSSSDEENDNKSVAPRVKRYVD
EIRFCDEDDEPKPARKAKKKSPAAAAENGGDLVKSVVKYRGVRQRPWGKFAAEIRDPS
SRTRLWLGTFATAEEAAIGYDRAAIRIKGHNAQTNFLTPPPSPTTEVLPETPVIDLETVSG
CDSARESQISLCSPTSVLRFSHNDETEYRTEPTEEQNPFFLPDLFRSGDYFWDSEITPDPLF
LDEFHQSLLPNINNNNNTVCDKDTNLSDSFPLGVIGDFSSWDVDEFFQDHLLDK*

>C3_AT_AT3G61630_AA // AtCRF6

MERRTRRVKFTENRTVTNVAATPSNGSPRLVRITVTDPFATDSSSDDDDNNNVTVVPRV
KRYVKEIRFCQGESSSSTAARKGKHKEEESVVVEDDVSTSVKPKKYRGVRQRPWGKFA
AEIRDPSSRTRIWLGTFVTAEEAAIAYDRAAIHLKGPKALTNFLTPPTPTPVIDLQTVSAC
DYGRDSRQSLHSPTSVLRFNVNEETEHEIEAIELSPERKSTVIKEEEESSAGLVFPDPYLLP

DLSLAGECFWDTEIAPDLLFLDEETKIQSTLLPNTEVSKQGENETEDFEFGLIDDFESSPW
DVDHFFDHHHHSFD*

>C78_AT_AT1G22985_AA // AtCRF7

MKRIVRISFTDMEATDSSSSEDESPPSSRRRGKKLVKEIVIDHSDPPEVGKTRFKIRIPASL
LAARNTTANKKKFRGVRQRPWGKWAAEIRCGRVKGRPERIWLGTFETAEEAALAYDN
AAIQLIGPDAPTNFGRPDVDSAVVKKQDSDASGGASEEVV*

>C78_AT_AT1G71130_AA // AtCRF8

MKRIIRISFTDAEATDSSSDEDTEERGGASQTRRRGKRLVKEIVIDPSDSADKLDVCKTRF
KIRIPAEFLKTAKTEKKYRGVRQRPWGKWVAEIRCGRGACKGRRDRLWLGTFNTAEEA
ALAYDNASIKLIGPHAPTNFGLPAENQEDKTVIGASEVARGA*

>C5_AT_AT1G49120_AA // AtCRF9

MISFREENIDLNLIKTISVICNDPDATDSSSDDESISGNNPRRQIKPKPPKRYVSKICVPTLI
KRYENVSNSTGNKAAGNRKTSSGFKGVRRRPWGKFAAEIRNPFEKKRKWLGTFPTEEE
AAEAYQKSKREFDERLGLVKQEKDLVDLTKPCGVRKPEEKEVTEKSNCKKVNKRIVTD
QKPFGCGYNADHEEEGVISKMLEDPLMTSSIADIFGDSAVEANDIWVDYNSVEFISIVDD
FKFDFVENDRVGKEKTFGFKIGDHTKVNQHAKIVSTNGDLFVDDLLDFDPLIDDFKLED
FPMDDLGLLGDPEDDDFSWFNGTTDWIDKFL*

>C5_AT_AT1G68550_AA // AtCRF10

MVAIRKEQSLSGVSSEIKKRAKRNTLSSLPQETQPLRKVRIIVNDPYATDDSSSDEEELKV
PKPRKMKRIVREINFPSMEVSEQPSESSSQDSTKTDGKIAVSASPAVPRKKPVGVRQRKW
GKWAAEIRDPIKKTRTWLGTFDTLEEAAKAYDAKKLEFDAIVAGNVSTTKRDVSSSETS
QCSRSSPVVPVEQDDTSASALTCVNNPDDVSTVAPTAPTPNVPAGGNKETLFDFDFTNL
QIPDFGFLAEEQQDLDFDCFLADDQFDDFGLLDDIQGFEDNGPSALPDFDFADVEDLQL
ADSSFGFLDQLAPINISCPLKSFAAS*

>C5_AT_At3g25890_AA // AtCRF11

MAERKKRSSIQTNKPNKKPMKKKPFQLNHLPGLSEDLKTMRKLRFVVNDPYATDYSSS
EEEERSQRRKRYVCEIDLPFAQAATQAESESSYCQESNNNGVSKTKISACSKKVLRSKAS
PVVGRSSTTVSKPVGVRQRKWGKWAAEIRHPITKVRTWLGTYETLEQAADAYATKKLE
FDALAAATSAASSVLSNESGSMISASGSSIDLDKKLVDSTLDQQAGESKKASFDFDFADL
QIPEMGCFIDDSFIPNACELDFLLTEENNNQMLDDYCGIDDLDIIGLECDGPSELPDYDFS
DVEIDLGLIGTTIDKYAFVDHIATTTPTPLNIACP*

>C5_AT_At1g25470_AA // AtCRF12

MKSFVKPERDSLLRTVRIVFTDPDATDDSSSSSDEWLPKPRKVKRFVHEITFLPQVSESSQ
DRSNAVKTPRRKSTRQFKYPVGVRPRPSGKFAAEILNPFTKTKKWLGTYETPAEAEKAY
VDKKVEYDALASSGSAVSSSVVTVTSQCLRSPTSASVSCVSADDLSKEKTSLNKDVAAS
GDSTTKEVFTTFDFSDVKIPDLRFLAAEEDSMVSNANGAELDFDCFLTDSNILLDDYSLL
ENDINFSRFENSLPSELPDCDFTEMEFQLDDFKFAYTDHLTTPPLGLV*

Supplemental Table 1: Full UniProt results

Clade	Run Type	Rank	E-value from MEME	Sites	UniProt ID	Gene Name	P-value from TOMTOM	GO-terms from Uniprot
I	OOPS	1	5E-144	88	Q9SKD0	BPC1	3.50E-09	Regulation of developmental process, regulation of transcription, response to ethylene, transcription
I	OOPS	1	5E-144	88	Q8L999	BPC6	5.14E-09	Regulation of transcription, response to ethylene, transcription
I	OOPS	1	5E-144	88	F4JUI3	BPC5	2.61E-08	Regulation of transcription, response to ethylene, transcription
I	OOPS	2	7E-168	88	Q9FGD6	DOF5.8	5.85E-10	Regulation of transcription, transcription
I	OOPS	2	7E-168	88	Q9SEZ3	CDF5	8.04E-10	Flower development, negative regulation of long- and short-day photoperiodism, reulgation of transcription, transcription
I	OOPS	2	7E-168	88	Q9M2U1	DOF3.6	4.27E-09	Regulation of transcription, transcription
I	OOPS	3	2E-81	88	Q9ZPX0	GATA20	2.99E-03	Cell differentiation, transcription, transcription regulation
I	OOPS	3	2E-81	88	C0SVG5	RVE5	1.36E-02	Response to abscisic acid, response to auxin, response to cadmium ion, response to ethylene, response to gibberllin, response to jasmonic acid, response to salicylic acid, response to salt stress, transcription
I	OOPS	3	2E-81	88	Q8H0W3	RVE6	1.53E-02	Regulation of circadian rhythm, response to abscisic acid, response to cadmium ion, response to ethylene, response to gibberllin, response to jasmonic acid, response to salicylic acid, response to salt stress, transcription
I	OOPS	4	1E-93	88	Q9FGD6	DOF5.8	3.55E-11	Regulation of transcription, transcription
I	OOPS	4	1E-93	88	Q9M2U1	DOF3.6	1.89E-10	Regulation of transcription, transcription
I	OOPS	4	1E-93	88	Q9LZ56	DOF5.1	2.37E-10	Regulation of transcription, transcription
I	OOPS	5	8E-104	88	P68350	DOF1.5	7.87E-05	Regulation of transcription, seed coat development, transcription
I	OOPS	5	8E-104	88	xxx	xxx	1.56E-04	xxx
I	OOPS	5	8E-104	88	O82155	DOF1.7	1.66E-04	Regulation of transcription, response to chitin, transcription
I	ZOOPS	1	8E-240	86	Q9SKD0	BPC1	8.24E-11	Regulation of developmental process, regulation of transcription, response to ethylene, transcription
I	ZOOPS	1	8E-240	86	F4JUI3	BPC5	2.90E-10	Regulation of transcription, response to ethylene, transcription
I	ZOOPS	1	8E-240	86	Q8L999	BPC6	1.32E-09	Regulation of transcription, response to ethylene, transcription
I	ZOOPS	2	6E-136	50	Q9ZPX0	GATA20	1.47E-03	Cell differentiation, transcription, transcription regulation
I	ZOOPS	2	6E-136	50	xxx	xxx	xxx	xxx
I	ZOOPS	2	6E-136	50	xxx	xxx	xxx	xxx
I	ZOOPS	3	1E-133	87	Q9M2U1	DOF3.6	2.82E-11	Regulation of transcription, transcription
I	ZOOPS	3	1E-133	87	Q9LZ56	DOF5.1	2.04E-10	Regulation of transcription, transcription
I	ZOOPS	3	1E-133	87	Q9SEZ3	CDF5	2.14E-10	Flower development, negative regulation of long- and short-day photoperiodism, reulgation of transcription, transcription
I	ZOOPS	4	4E-132	83	Q39088	DOF3.4	6.37E-05	Cell wall modification, positive regulation of cell cycle, positive regulation of transcription, response to auxin, response to salicylic acid, transcription
I	ZOOPS	4	4E-132	83	Q8L999	BPC6	7.72E-05	Regulation of transcription, response to ethylene, transcription
I	ZOOPS	4	4E-132	83	Q9FGD6	DOF5.8	1.09E-04	Regulation of transcription, transcription
I	ZOOPS	5	2E-107	25	A9RZ73	xxx	9.69E-06	predicted protein
I	ZOOPS	5	2E-107	25	Q9CAA4	BIM2	5.79E-05	Positive brassinosteroid signaling protein, regulation of transcription, transcription
I	ZOOPS	5	2E-107	25	Q9M7Q5	ABF1	2.06E-04	Abscisic acid-activated signaling pathway, transcription

Clade	Run Type	Rank	E-value from MEME	Sites	UniProt ID	Gene Name	P-value from TOMTOM	GO-terms from Uniprot
II	OOPS	1	2E-215	85	Q9ZPX0	GATA20	2.20E-03	Cell differentiation, transcription, transcription regulation
II	OOPS	1	2E-215	85	Q6R053	MYB56	6.15E-03	Brassinosteroid mediated signaling pathway, cell differentiation, cellular response to brassinosteroid stimulus, endothelial cell proliferation, integument development, negative regulation of cell division, negative resulation of long-day photoperiodism, positive regulation of transcription, quiescent center organization, regulation of seed growth, regulation of transcription, regulation of transcription from RNA polymerase II promoter, transcription
II	OOPS	1	2E-215	85	Q9LSL6	DOF5.7	6.78E-03	Guard cell differentiation, positive regulation of transcription, regulation of cell wall pectin metabolic process, regulation of transcription, stomatal movement
II	OOPS	2	4E-193	85	A9RZ73	PHYPADRAFT_72483	9.24E-06	Predicted protein
II	OOPS	2	4E-193	85	Q9CAA4	BIM2	5.75E-05	Transcription, transcription regulation
II	OOPS	2	4E-193	85	Q9LT89	TCP19	8.85E-05	Regulation of defense response, regulation of transcription, transcription
II	OOPS	3	1E-207	85	Q9SKD0	BPC1	5.52E-16	Regulation of developmental process, regulation of transcription, response to ethylene, transcription
II	OOPS	3	1E-207	85	F4JUI3	BPC5	5.55E-15	Regulation of transcription, response to ethylene, transcription
II	OOPS	3	1E-207	85	Q8L999	BPC6	1.11E-13	Regulation of transcription, response to ethylene, transcription
II	OOPS	4	1E-121	85	P93007	ERF112	4.48E-03	Ethylene-activated signaling pathway, transcription
II	OOPS	4	1E-121	85	O80339	ERF3	4.52E-03	Defense response, ethylene-activated signaling pathway, negative regulation of ethylene-activated signaling pathway, transcription
II	OOPS	4	1E-121	85	O24646	HY5	4.86E-03	Abscisic acid-activated signaling pathway, gibberellic acid mediated signaling pathway, positive gravitropism, positive regulation of anthyocyanin metabolic process, positive regulation of circadian rhythm, red/far-red light phototransduction, red/far-red light signaling pathway, regulation of absciscic acid-activated signaling pathway, regulation of photomorphogeneiss, response to abscisic acid, response to far red light, response to karrikin, response to red light, response to UV-B, transcription
II	OOPS	5	3E-68	85	P92948	CDC5	6.53E-04	Cell cycle, cell differentiation, defense response signaling pathway, defense response to bacterium, defense response to fungus, DNA repair, innate immune response, mRNA processing, regulation of transcription, regulation of transcription from RNA polymerase II promoter, RNA splicing, transcription
II	OOPS	5	3E-68	85	O22900	WRKY23	1.60E-03	Response to auxin, response to nematode, transcription
II	OOPS	5	3E-68	85	Q9SKD9	WRKY46	3.27E-03	Lateral root development, regulation of transcription, response to chitin, transcription
II	ZOOPS	1	2E-280	44	A9RZ73	PHYPADRAFT_72483	4.70E-06	Predicted protein
II	ZOOPS	1	2E-280	44	Q9CAA4	BIM2	3.77E-05	Transcription, transcription regulation
II	ZOOPS	1	2E-280	44	Q9LEZ3	BIM1	6.81E-05	Regulation of transcription, transcription
II	ZOOPS	2	1E-243	70	Q9ZPX0	GATA20	1.70E-03	Cell differentiation, transcription, transcription regulation
II	ZOOPS	2	1E-243	70	O64647	TCP9	7.16E-03	Negative regulation of leaf senescence, regulation of cell size, regulation of transcription, root development, transcription
II	ZOOPS	2	1E-243	70	Q9LSL6	DOF5.7	9.78E-03	Guard cell differentiation, positive regulation of transcription, regulation of cell wall pectin metabolic process, regulation of transcription, stomatal movement
II	ZOOPS	3	1E-207	85	Q9SKD0	BPC1	5.52E-16	Regulation of developmental process, regulation of transcription, response to ethylene, transcription
II	ZOOPS	3	1E-207	85	F4JUI3	BPC5	5.55E-15	Regulation of transcription, response to ethylene, transcription
II	ZOOPS	3	1E-207	85	Q8L999	BPC6	1.11E-13	Regulation of transcription, response to ethylene, transcription
II	ZOOPS	4	1E-160	53	Q38Q40	ERF112	3.53E-03	Ethylene-activated signaling pathway, transcription
II	ZOOPS	4	1E-160	53	Q9LND1	ERF094	4.77E-03	Ethylene-activated signaling pathway, jasmonic acid and ethylene-dependent systemic resistance, response to ethylene, response to jasmonic acid, transcription
II	ZOOPS	4	1E-160	53	P93007	ERF112	7.09E-03	Ethylene-activated signaling pathway, transcription
II	ZOOPS	5	2E-133	36	P92948	CDC5	6.82E-04	Cell cycle, cell differentiation, defense response signaling pathway, defense response to bacterium, defense response to fungus, DNA repair, innate immune response, mRNA processing, regulation of transcription, regulation of transcription from RNA polymerase II promoter, RNA splicing, transcription
II	ZOOPS	5	2E-133	36	A9TN45	PHYPADRAFT_48267	1.48E-03	Predicted protein
II	ZOOPS	5	2E-133	36	O22900	WRKY23	1.48E-03	Response to auxin, response to nematode, transcription

Clade	Run Type	Rank	E-value from MEME	Sites	UniProt ID	Gene Name	P-value from TOMTOM	GO-terms from Uniprot
III	OOPS	1	1E-101	46	Q8L999	BPC6	1.91E-15	Regulation of transcription, response to ethylene, transcription
III	OOPS	1	1E-101	46	Q9SKD0	BPC1	5.62E-13	Regulation of developmental process, regulation of transcription, response to ethylene, transcription
III	OOPS	1	1E-101	46	F4JUI3	BPC5	1.70E-11	Regulation of transcription, response to ethylene, transcription
III	OOPS	2	2E-86	46	Q01593	ABI3	1.97E-03	Abscisic acid-activated signaling pathway, embryo development ending in seed dormancy, mitochondira-nucleus signaling pathway, plastid organization, positive regulation of transcription, response to abscisic acid, response to auxin, transcription
III	OOPS	2	2E-86	46	D7L3Y2	ARALYDRAFT_897773	3.77E-03	Predicted protein; cell differentiation, cotyledon morphogenesis, positive regulation of development, heterochronic, regulation of transcription, transcription
III	OOPS	2	2E-86	46	Q9LEZ9	TCP17	5.91E-03	Cell differentiation, leaf morphogenesis, positive regulation of development, heterochronic, regulation of transcription, transcription
III	OOPS	3	4E-79	46	Q9ZWJ9	ARR2	3.69E-03	Cellular response to cytokinin stimulus, cytokinin-activated signaling pathway, ethylene-activated signaling pathway, leaf senescence, regulation of root meristem growth, regulation of seed growth, regulation of stomatal movement, response to cytokinin, response to etylene, root development, transcription
III	OOPS	3	4E-79	46	Q9SWG3	FAR1	1.17E-02	Far-red light signaling pathway, positive regulation of circadian rhythm, positive regulation of transcription, red/far-red phototransduction, red/far-red light signaling pathway, response to red/far red light
III	OOPS	3	4E-79	46	Q8L9Y3	ARR14	1.29E-02	Regulation of transcription, transcription
III	OOPS	4	8E-69	46	Q9FX67	HRS1	1.32E-03	Cellular response to nitrogen compound, cellular response to phosphate starvation, negative regulation of abscisic acid-activated signaling pathway, primary root development, regulation of response to nutrient levels, regulation of transcription, transcription
III	OOPS	4	8E-69	46	Q8L9Y3	ARR14	7.03E-03	Cytokinin-activated signaling pathway, transcription
III	OOPS	4	8E-69	46	Q8VZS3	HHO2	8.63E-03	Phosphate ion homeostasis, regulation of transcription, response to abscisic acid, transcription
III	OOPS	5	9E-34	46	Q9M2U1	OBF3/DOF3.6	4.65E-09	Regulation of transcription, transcription
III	OOPS	5	9E-34	46	Q9SEZ3	CDF5	8.03E-09	Flower development, negative regulation of long-/short-day photoperiodism, regulation of transcription, transcription
III	OOPS	5	9E-34	46	Q9LZ56	DOF5.1	8.85E-08	Regulation of transcription, transcription
III	ZOOPS	1	2E-107	30	Q8L9Y3	ARR14	1.05E-02	Regulation of transcription, transcription
III	ZOOPS	1	2E-107	30	Q9ZWJ9	ARR2	1.29E-02	Cellular response to cytokinin stimulus, cytokinin-activated signaling pathway, ethylene-activated signaling pathway, leaf senescence, regulation of root meristem growth, regulation of seed growth, regulation of stomatal movement, response to cytokinin, response to etylene, root development, transcription
III	ZOOPS	1	2E-107	30	Q9SWG3	FAR1	1.37E-02	Far-red light signaling pathway, positive regulation of circadian rhythm, positive regulation of transcription, red/far-red phototransduction, red/far-red light signaling pathway, response to red/far red light
III	ZOOPS	2	1E-108	42	Q9SKD0	BPC1	4.35E-17	Regulation of developmental process, regulation of transcription, response to ethylene, transcription
III	ZOOPS	2	1E-108	42	F4JUI3	BPC5	1.21E-15	Regulation of transcription, response to ethylene, transcription
III	ZOOPS	2	1E-108	42	Q8L999	BPC6	6.94E-15	Regulation of transcription, response to ethylene, transcription
III	ZOOPS	3	8E-107	34	Q01593	ABI3	4.32E-03	Abscisic acid-activated signaling pathway, embryo development ending in seed dormancy, mitochondira-nucleus signaling pathway, plastid organization, positive regulation of transcription, response to abscisic acid, response to auxin, transcription
III	ZOOPS	3	8E-107	34	D7L3Y2	ARALYDRAFT_897773	6.97E-03	Predicted protein; cell differentiation, cotyledon morphogenesis, positive regulation of development, heterochronic, regulation of transcription, transcription
III	ZOOPS	3	8E-107	34	Q9LEZ9	TCP17	8.37E-03	Cell differentiation, leaf morphogenesis, positive regulation of development, heterochronic, regulation of transcription, transcription
III	ZOOPS	4	5E-98	30	Q9FX67	HRS1	1.82E-03	Cellular response to nitrogen compound, cellular response to phosphate starvation, negative regulation of abscisic acid-activated signaling pathway, primary root development, regulation of response to nutrient levels, regulation of transcription, transcription
III	ZOOPS	4	5E-98	30	Q8VZS3	HHO2	5.72E-03	Phosphate ion homeostasis, regulation of transcription, response to abscisic acid, transcription
III	ZOOPS	4	5E-98	30	Q8L9Y3	ARR14	9.18E-03	Regulation of transcription, transcription
III	ZOOPS	5	5E-57	30	Q9SAH7	WRKY40	3.42E-05	Defense response to bacterium, defense response to fungus, negative regulation of transcription, regulation of defense response, response to chitin, reponse to molecule of bacterial origin, response to salicylic acid, response to wounding, transcription
III	ZOOPS	5	5E-57	30	Q9LX86	F12M12_40	6.17E-04	Regulation of transcription, transcription
III	ZOOPS	5	5E-57	30	Q8VWQ5	WRKY50	7.17E-04	Defense response to fungus, jasmonic acid mediated signaling pathway, transcription

Clade	Run Type	Rank	E-value from MEME	Sites	UniProt ID	Gene Name	P-value from TOMTOM	GO-terms from Uniprot
IV	OOPS	1	1E-35	20	Q9SKD0	BPC1	2.57E-12	Regulation of developmental process, regulation of transcription, response to ethylene, transcription
IV	OOPS	1	1E-35	20	F4JUI3	BPC5	1.88E-10	Regulation of transcription, response to ethylene, transcription
IV	OOPS	1	1E-35	20	Q8L999	BPC6	5.55E-10	Regulation of transcription, response to ethylene, transcription
IV	OOPS	2	3E-21	20	Q9FNY0	E2FA	6.34E-04	Cell cycle, positive regulation of meitoic cell cycle, positive regulation of transcription, transcription
IV	OOPS	2	3E-21	20	Q9SW63	RAP2-10	1.54E-03	Ethylene-activated signaling pathway, transcription
IV	OOPS	2	3E-21	20	Q9FGV1	ARF8	9.17E-03	Auxin-activated signaling pathway, flower development, response to auxin, transcription
IV	OOPS	3	5E-10	20	Q9SKD0	BPC1	1.69E-04	Regulation of developmental process, regulation of transcription, response to ethylene, transcription
IV	OOPS	3	5E-10	20	Q8L999	BPC6	2.22E-04	Regulation of transcription, response to ethylene, transcription
IV	OOPS	3	5E-10	20	XXX	RAMOSA1	2.22E-04	
IV	OOPS	4	2E-04	20	Q9M2U1	OBP3/DOF3.6	5.83E-08	Regulation of transcription, transcription
IV	OOPS	4	2E-04	20	Q9FGD6	DOF5.8	8.51E-08	Regulation of transcription, transcription
IV	OOPS	4	2E-04	20	Q9SEZ3	CDF5	3.14E-07	Flower development, negative regulation of long-/short-day photoperiodism, regulation of transcription, transcription
IV	OOPS	5	0.002	20	Q1PFR7	LEC2	1.55E-03	Embryo development ending in seed dormancy, positive regulation of auxin biosynthesis process, positive regulation of transcription, seed maturation, seed oilbody biogenesis, somatic embryogenesis, transcription
IV	OOPS	5	0.002	20	Q01593	ABI3	1.75E-03	Abscisic acid-activated signaling pathway, embryo development ending in seed dormancy, mitochondira-nucleus signaling pathway, plastid organization, positive regulation of transcription, response to abscisic acid, response to auxin, transcription
IV	OOPS	5	0.002	20	Q8LFV3	CDF3	7.04E-03	Flower development, regulation of transcription, transcription
IV	ZOOPS	1	1E-35	20	Q9SKD0	BPC1	2.57E-12	Regulation of developmental process, regulation of transcription, response to ethylene, transcription
IV	ZOOPS	1	1E-35	20	F4JUI3	BPC5	1.88E-10	Regulation of transcription, response to ethylene, transcription
IV	ZOOPS	1	1E-35	20	Q8L999	BPC6	5.55E-10	Regulation of transcription, response to ethylene, transcription
IV	ZOOPS	2	5E-23	19	Q9FNY0	E2FA	8.09E-04	Cell cycle, positive regulation of meitoic cell cycle, positive regulation of transcription, transcription
IV	ZOOPS	2	5E-23	19	Q9SW63	RAP210	9.62E-04	Ethylene-activated signaling pathway, transcription
IV	ZOOPS	2	5E-23	19	Q9FJ93	DREB1D	4.41E-03	Abscisic acid-activated signaling pathway, glucosinolate metabolic process, transcription
IV	ZOOPS	3	1E-11	20	Q8L999	BPC6	7.81E-05	Regulation of transcription, response to ethylene, transcription
IV	ZOOPS	3	1E-11	20	Q9SKD0	BPC1	9.77E-05	Regulation of developmental process, regulation of transcription, response to ethylene, transcription
IV	ZOOPS	3	1E-11	20	Q9LX86	F12M12_40	1.37E-04	Regulation of transcription, response to chitin
IV	ZOOPS	4	2E-12	10	Q01593	ABI3	2.39E-03	Abscisic acid-activated signaling pathway, embryo development ending in seed dormancy, mitochondira-nucleus signaling pathway, plastid organization, positive regulation of transcription, response to abscisic acid, response to auxin, transcription
IV	ZOOPS	4	2E-12	10	Q1PFR7	LEC2	3.26E-03	Embryo development ending in seed dormancy, positive regulation of auxin biosynthesis process, positive regulation of transcription, seed maturation, seed oilbody biogenesis, somatic embryogenesis, transcription
IV	ZOOPS	4	2E-12	10	Q8LFV3	CDF3	4.93E-03	Flower development, regulation of transcription, transcription
IV	ZOOPS	5	5E-11	7	Q9M2Y9	RAX3	9.69E-03	Cell differentiation, multicellular organism development, regulation of transcription from RNA polymerase II promoter, response to salicylic acid, transcription
IV	ZOOPS	5	5E-11	7	Q8VWG0	bZIP52	1.87E-02	Regulation of transcription
IV	ZOOPS	5	5E-11	7	XXX	XXX	XXX	

Clade	Run Type	Rank	E-value from MEME	Sites	UniProt ID	Gene Name	P-value from TOMTOM	GO-terms from Uniprot
V	OOPS	1	1E-231	66	Q1PFR7	LEC2	1.98E-03	Embryo development ending in seed dormancy, positive regulation of auxin biosynthesis process, positive regulation of transcription, seed maturation, seed oilbody biogenesis, somatic embryogenesis, transcription
V	OOPS	1	1E-231	66	Q9ZPY0	DOF2.5	1.07E-02	Cellular response to red light, cellular response to water stimulus, positive regulation of gibberellin biosynthetic process, positive regulation of seed germination, red light signaling pathway, response to cold, response to light stimulus, seed germination, transcription
V	OOPS	1	1E-231	66	O80928	DOF2.4	1.36E-02	Regulation of trascription, transcription
V	OOPS	2	1E-170	66	Q5DW98	PEND	5.40E-03	Predicted protein
V	OOPS	2	1E-170	66	Q9ZUL3	IDD5	1.35E-02	Positive regulation of starch synthase SS4, development of chloroplast and startch granules, regulation of transcription, transcription
V	OOPS	2	1E-170	66	Q9FM03	DOF5.6	1.49E-02	Phloem or xylem histogenesis, positive regulation of transcription, procambium histogenesis, regulation of transcription, transcription
V	OOPS	3	2E-174	66	Q9SGJ6	DREB1E	2.95E-05	Regulation of gibberellin biosynthesis process, regulation of timing of transcition from vegetative to reproductive phase, transcription
V	OOPS	3	2E-174	66	Q9SYS6	DRE1C	9.59E-05	Cold acclimation, response to cold, transcription
V	OOPS	3	2E-174	66	Q9FGV1	ARF8	1.77E-04	Auxin-activated signaling pathway, flower development, response to auxin, transcription
V	OOPS	4	2E-130	66	O82155	DOF1.7	2.09E-09	Regulation of transcription, response to chitin, transcription
V	OOPS	4	2E-130	66	Q9FGD6	DOF5.8	5.03E-09	Regulation of transcription, transcription
V	OOPS	4	2E-130	66	Q39088	DOF3.4	6.98E-09	Cell wall modification, positive regulation of cell cycle, positive regulation of transcription, response to auxin, response to salicylic acid, transcription
V	OOPS	5	4E-97	66	O49397	ARR10	8.49E-03	Cellular response to cytokinin stimulus, cytokinin-activated signaling pathway, maintenance of shoot apical meristem identity, primary root development, regulation of anthocyanin metabolic process, regulation of chlorophyll biosynthetic process, regulation of cytokinin-activated signaling pathway, regulation of root meristem growth, regulation of seed growth, response to cytokinin, response to water deprevivation, root development, shoot system development, transcription
V	OOPS	5	4E-97	66	Q9FJW5	TRB2	1.24E-02	Nucleosome assembly, response to abscisic acid, response to auxin, cadmium ion, response to ethylene, response to gibberellin, response to jasmonic acid, response to salicylic acid, response to salt stress, telomeric loop formation, transcription
V	OOPS	5	4E-97	66	Q6DBP8	GATA11	1.89E-02	Cell differentiation, regulation of transcription, transcription
V	ZOOPS	1	2E-279	47	Q1PFR7	LEC2	4.67E-03	Embryo development ending in seed dormancy, positive regulation of auxin biosynthesis process, positive regulation of transcription, seed maturation, seed oilbody biogenesis, somatic embryogenesis, transcription
V	ZOOPS	1	2E-279	47	Q9ZPY0	DOF2.5	1.33E-02	Cellular response to red light, cellular response to water stimulus, positive regulation of gibberellin biosynthetic process, positive regulation of seed germination, red light signaling pathway, response to cold, response to light stimulus, seed germination, transcription
V	ZOOPS	1	2E-279	47	O22456	Sep3	1.36E-02	Cell fate specification, flower development, plant ovule development, positive regulation of transcription from RNA polymerase II promoter, specification of floral organ identity, specification of floral organ number, transcription
V	ZOOPS	2	9E-207	51	Q5DW98	PEND	5.42E-03	Predicted protein
V	ZOOPS	2	9E-207	51	XXX	XXX	XXX	
V	ZOOPS	2	9E-207	51	XXX	XXX	XXX	
V	ZOOPS	3	3E-194	46	Q9SGJ6	DREB1E	7.57E-06	Regulation of gibberellin biosynthesis process, regulation of timing of transcition from vegetative to reproductive phase, transcription
V	ZOOPS	3	3E-194	46	Q9SYS6	DRE1C	6.87E-05	Cold acclimation, response to cold, transcription
V	ZOOPS	3	3E-194	46	Q9FGV1	ARF8	1.87E-04	Auxin-activated signaling pathway, flower development, response to auxin, transcription
V	ZOOPS	4	3E-135	66	O82155	DOF1.7	2.80E-09	Regulation of transcription, response to chitin, transcription
V	ZOOPS	4	3E-135	66	Q9FGD6	DOF5.8	3.83E-09	Regulation of transcription, transcription
V	ZOOPS	4	3E-135	66	Q39088	DOF3.4	1.50E-08	Cell wall modification, positive regulation of cell cycle, positive regulation of transcription, response to auxin, response to salicylic acid, transcription
V	ZOOPS	5	4E-124	37	P29383	AGL3	5.35E-03	Carpel development, cell differentiation, maintenance of floral meristem identity, petal development, positive regulation of transcription from RNA polymerase II promoter, sepal development, stamen development, transcription
V	ZOOPS	5	4E-124	37	Q38847	AGL15	1.20E-02	Cellular response to auxin stimulus, embryo development ending in seed dormancy, floral organ abscission, fruit abscission, fruit dehiscence, gibberellin catabolic process, negative regulation of floral organ abscission, negative regulation of flower development, negative regulation of gene expression, negative regulation of seed maturation, negative regulation of short-day photoperiodism, negative/positive regulation of transcription, positive regulation of transcription from RNA polymerase II promoter, somatic embryogenesis, transcription
V	ZOOPS	5	4E-124	37	Q9ZUL3	IDD5	1.88E-02	Positive regulation of starch synthase SS4, development of chloroplast and startch granules, regulation of transcription, transcription

Clade	Run Type	Rank	E-value from MEME	Sites	UniProt ID	Gene Name	P-value from TOMTOM	GO-terms from Uniprot
Un	OOPS	1	3E-23	20	Q9M2U1	OBP3/DOF3.6	1.60E-10	Regulation of transcription, transcription
Un	OOPS	1	3E-23	20	Q9FGD6	DOF5.8	7.64E-09	Regulation of transcription, transcription
Un	OOPS	1	3E-23	20	Q9SEZ3	CDF5	1.40E-08	Flower development, negative regulation of long-/short-day photoperiodism, regulation of transcription, transcription
Un	OOPS	2	1E-17	20	F4JRB0	HHO5	3.78E-04	Floral organ formation, negative regulation of gene expression, regulation of transcription, specification of plant organ identity, transcription
Un	OOPS	2	1E-17	20	F4JRB0	HHO5	1.59E-03	Floral organ formation, negative regulation of gene expression, regulation of transcription, specification of plant organ identity, transcription
Un	OOPS	2	1E-17	20	Q9FGD6	DOF5.8	3.50E-03	Regulation of transcription, transcription
Un	OOPS	3	3E-20	20	Q9FFH3	NUC	2.91E-04	Asymmetic cell division, leaf senescence, flowering photoperiodism, positive regulation of transcription, regulation of timining of transcition from vegetative to reproductive phase, transcription
Un	OOPS	3	3E-20	20	Q9ZWA6	MGP	3.26E-04	Asymmetic cell division, regulation of transcription, transcription
Un	OOPS	3	3E-20	20	O80917	DREB2E	4.40E-04	Abscisic acid-activated signaling pathway, cellular response to heat, positive regulation of transcrition, response to salt stress, response to water deprivation, transcription
Un	OOPS	4	1E-04	20	Q9FGD6	DOF5.8	2.74E-04	Regulation of transcription, transcription
Un	OOPS	4	1E-04	20	Q39088	DOF3.4	3.52E-04	Cell wall modification, positive regulation of cell cycle, positive regulation of transcription, response to auxin, response to salicyilc acid, transcription
Un	OOPS	4	1E-04	20	Q9LZ56	DOF5.1	5.13E-04	Regulation of transcription, transcription
Un	OOPS	5	7E-04	20	Q9SEZ3	CDF5	6.49E-04	Flower development, negative regulation of long-/short-day photoperiodism, regulation of transcription, transcription
Un	OOPS	5	7E-04	20	Q9FKA0	NAC92	7.65E-04	Lateral root development, leaf senescence, positive regulation of age-related resistance, positive regulation of DNA binding transcription factor activity, positive regulation of leaf senescence, positive regulation of programmed cell death, regulation of gene expression, regulation of seed germination, response to abscisic acid, response to auxin, response to etylene, response to hydrogen peroxide, response to oxidative stress, response to salt, response to salt stress, stress-induced premature senescence, transcription
Un	OOPS	5	7E-04	20	Q9S7L2	MYB98	1.56E-03	Embryo sac development, pollen tube guidance, regulation of embryo sac central cell differentiation, regulation of synergid differentiation, regulation of transcription, regulation of transcription from RNA polymerase II promoter, transcription
Un	ZOOPS	1	3E-23	20	Q9M2U1	OBP3/DOF3.6	1.60E-10	Regulation of transcription, transcription
Un	ZOOPS	1	3E-23	20	Q9LZ56	DOF5.1	7.64E-09	Regulation of transcription, transcription
Un	ZOOPS	1	3E-23	20	Q9SEZ3	CDF5	1.40E-08	Flower development, negative regulation of long-/short-day photoperiodism, regulation of transcription, transcription
Un	ZOOPS	2	2E-22	12	Q38Q39	ERF027	1.20E-03	Ethylene-activated signaling pathway, glucosinolate metabolic process, transcription
Un	ZOOPS	2	2E-22	12	Q38914	ANT	2.35E-03	Cell differentiation, flower development, glucosinolate metabolic process, maintenance of shoot apical meristem identity, regulation of cell proliferation, transcription
Un	ZOOPS	2	2E-22	12	Q8VZP4	GATA10	2.37E-03	Cell differentiation, regulation of transcription, transcription
Un	ZOOPS	3	8E-20	15	F4JRB0	HHO5	5.56E-04	Floral organ formation, negative regulation of gene expression, regulation of transcription, specification of plant organ identity, transcription
Un	ZOOPS	3	8E-20	15	Q9FGD6	DOF5.8	8.57E-04	Regulation of transcription, transcription
Un	ZOOPS	3	8E-20	15	F4JRB0	HHO5	1.56E-03	Floral organ formation, negative regulation of gene expression, regulation of transcription, specification of plant organ identity, transcription
Un	ZOOPS	4	2E-20	19	Q9ZWA6	MGP	2.35E-04	Asymmetic cell division, leaf senescence, flowering photoperiodism, positive regulation of transcription, regulation of timining of transcition from vegetative to reproductive phase, transcription
Un	ZOOPS	4	2E-20	19	Q9FFH3	NUC	2.64E-04	Asymmetic cell division, regulation of transcription, transcription
Un	ZOOPS	4	2E-20	19	O80917	DREB19	2.81E-04	Abscisic acid-activated signaling pathway, cellular response to heat, positive regulation of transcrition, response to salt stress, response to water deprivation, transcription
Un	ZOOPS	5	7E-21	12	Q39088	DOF2.2	1.96E-05	Regulation of transcription, transcription
Un	ZOOPS	5	7E-21	12	Q9ZV33	DOF3.4	1.21E-04	Cell wall modification, positive regulation of cell cycle, positive regulation of transcription, response to auxin, response to salicyilc acid, transcription
Un	ZOOPS	5	7E-21	12	Q9FGD6	DOF5.8	1.24E-04	Regulation of transcription, transcription

Clade	Run Type	Rank	E-value from MEME	Sites	UniProt ID	Gene Name	P-value from TOMTOM	GO-terms from Uniprot
ALL	OOPS	1	4.4e-575	346	Q9SKD0	BPC1	6.92E-13	Regulation of developmental process, regulation of transcription, response to ethylene, transcription
ALL	OOPS	1	4.4e-575	346	F4JUI3	BPC5	3.31E-11	Regulation of transcription, response to ethylene, transcription
ALL	OOPS	1	4.4e-575	346	Q8L999	BPC6	5.00E-11	Regulation of transcription, response to ethylene, transcription
ALL	OOPS	2	8.8e-411	346	Q9M2U1	DOF3.6	5.05E-11	Regulation of transcription, transcription
ALL	OOPS	2	8.8e-411	346	Q9SEZ3	CDF5	1.02E-10	Flower development, negative regulation of long-/short-day photoperiodism, regulation of transcription, transcription
ALL	OOPS	2	8.8e-411	346	Q9LZ56	DOF5.1	1.17E-10	Regulation of transcription, transcription
ALL	OOPS	3	######	346	Q9SKD0	BPC1	3.87E-11	Regulation of developmental process, regulation of transcription, response to ethylene, transcription
ALL	OOPS	3	######	346	XXX	RAMOSA1	8.64E-11	Predicted protein
ALL	OOPS	3	######	346	Q8L999	BPC6	1.01E-10	Regulation of transcription, response to ethylene, transcription
ALL	OOPS	4	######	346	Q9FGD6	DOF5.8	2.37E-11	Regulation of transcription, transcription
ALL	OOPS	4	######	346	Q9M2U1	DOF3.6	4.53E-10	Regulation of transcription, transcription
ALL	OOPS	4	######	346	Q9LZ56	DOF5.1	1.73E-09	Regulation of transcription, transcription
ALL	OOPS	5	######	346	Q9LSL6	DOF5.7	4.12E-03	Guard cell differentiation, positive regulation of transcription, regulation of cell wall pectin metabolic process, regulation of transcription, stomatal movement
ALL	OOPS	5	######	346	Q6R053	MYB56	4.21E-03	Brassinosteroid mediated signaling pathway, cell differentiation, cellular response to brassinosteroid stimulus, endothelial cell proliferation, integument development, negative regulation of cell division, negative resulation of long-day photoperiodism, positive regulation of transcription, quiescent center organization, regulation of seed growth, regulation of transcription, regulation of transcription from RNA polymerase II promoter, transcription
ALL	OOPS	5	######	346	Q9ZPX0	GATA20	8.07E-03	Cell differentiation, regulation of transcription
ALL	ZOOPS	1	2.7e-527	346	Q9SKD0	BPC1	4.07E-11	Regulation of developmental process, regulation of transcription, response to ethylene, transcription
ALL	ZOOPS	1	2.7e-527	346	Q8L999	BPC6	6.69E-11	Regulation of transcription, response to ethylene, transcription
ALL	ZOOPS	1	2.7e-527	346	xxx	RAMOSA1	1.34E-10	
ALL	ZOOPS	2	1.7e-333	345	Q9M2U1	DOF3.6	4.25E-10	Regulation of transcription, transcription
ALL	ZOOPS	2	1.7e-334	345	Q9SEZ3	CDF5	2.76E-09	Flower development, negative regulation of long-/short-day photoperiodism, regulation of transcription, transcription
ALL	ZOOPS	2	1.7e-335	345	Q9FGD6	DOF5.8	5.73E-09	Regulation of transcription, transcription
ALL	ZOOPS	3	######	137	Q9ZPX0	GATA20	1.32E-03	Cell differentiation, regulation of transcription
ALL	ZOOPS	3	3E-245	137				
ALL	ZOOPS	3	3E-245	137				
ALL	ZOOPS	4	3E-217	295		RAMOSA1	9.5E-10	
ALL	ZOOPS	4	######	295	Q9SKD0	BPC1	2.14E-09	Regulation of developmental process, regulation of transcription, response to ethylene, transcription
ALL	ZOOPS	4	######	295	Q8L999	BPC6	6.50E-09	Regulation of transcription, response to ethylene, transcription
ALL	ZOOPS	5	######	117	Q9SLH1	MYB81	6.63E-03	Cell differentiation, regulation of transcription from RNA polymerase II promoter
ALL	ZOOPS	5	######	117	P42736	RAP2-3	6.77E-03	Cell death, ethylene-activated signaling pathway, heat acclimation, positive regulation of transcription, response to cytokinin, response to ethylene, response to jasmonic acid, response to other organism, transcription
ALL	ZOOPS	5	######	117	P93007	ERF112	8.23E-03	Ethylene-activated signaling pathway, transcription

Clade	Run Type	Rank	E-value from MEME	Sites	UniProt ID	Gene Name	P-value from TOMTOM	GO-terms from Uniprot
I-B	OOPS	1	1.6e-073	15	Q9FGT7	ARR18	4.26E-03	Cytokinin signaling pathway, Transcription, Transcription regulation, Two-component regulatory system
I-B	OOPS	1	1.6e-073	15	Q9FPE8	HHO3	1.70E-02	Transcription, Transcription regulation; Probable transcription factor involved in phosphate signaling in roots.
I-B	OOPS	1	1.6e-073	15	Q9ZPX0	GATA20	4.56E-03	Transcription, Transcription regulation; Transcriptional regulator that specifically binds 5'-GATA-3' or 5'-GAT-3' motifs within gene promoters
I-B	OOPS	2	3.5e-050	15	O49687	MYC4	7.32E-03	Anthocyanin-containing compound biosynthetic process, jasmonic acid gene regulation, defense response, positive regulation of transcription, regulation of transcription, transcription
I-B	OOPS	2	3.5e-050	15	Q9LSL6	DOF5.7	5.02E-03	Guard cell differentiation, positive regulation of transcription, regulation of cell wall pectin metabolic process, regulation of transcription, stomatal movement
I-B	OOPS	2	3.5e-050	15	Q9SEZ3	CDF5	7.14E-03	Flower development, negative regulation of long-/short-day photoperiodism, regulation of transcription, transcription
I-B	OOPS	3	6.6e-048	15	Q39088	DOF3.4	1.52E-03	Cell wall modification, positive regulation of cell cycle, positive regulation of transcription, response to auxin, response to salicyilc acid, transcription
I-B	OOPS	3	6.6e-048	15	Q9LZ56	DOF5.1	3.45E-03	Regulation of transcription, transcription
I-B	OOPS	3	6.6e-048	15	Q9ZV33	DOF2.2	9.24E-04	Regulation of transcription, transcription
I-B	OOPS	4	2.4e-037	15	C0SVS4	PHL11	2.92E-04	Transcription, Transcription regulation
I-B	OOPS	4	2.4e-037	15	F4IPE3	SGR5	4.78E-04	Circumnution, detection of gravity, floral organ morphogenesis, gravitropism, leaf morphogenesis, positive regulation of auxin biosynthetic process, regulation of auxin polar transport, regulation of startch metabolic process, regulation of transcription
I-B	OOPS	4	2.4e-037	15	Q9LSL6	DOF5.7	5.02E-03	Guard cell differentiation, positive regulation of transcription, regulation of cell wall pectin metabolic process, regulation of transcription, stomatal movement
I-B	OOPS	5	5.0e-032	15	F4IPE3	SGR5	4.78E-04	Circumnution, detection of gravity, floral organ morphogenesis, gravitropism, leaf morphogenesis, positive regulation of auxin biosynthetic process, regulation of auxin polar transport, regulation of startch metabolic process, regulation of transcription
I-B	OOPS	5	5.0e-032	15	F4JN35	NTL9	1.12E-03	Cellular response to osmotic stress, negative regulation of transcription, positive regulation of defense responser to bacterium, regulation of defense response, regulation of transcription, transcription
I-B	OOPS	5	5.0e-032	15	Q8GYC1	IDD4	2.47E-03	Assymetic cell division, root development, transcription
II-B	OOPS	1	2.3e-153	24	Q01593	ABI3	6.16E-03	Abscisic acid-activated signaling pathway, embryo development, mitochondira-nucleus signaling pathway, plastid organization, postive regulation of transcription, response to abscisic acid, response to auxin, transcription
II-B	OOPS	1	2.3e-153	24	Q38Q40	ERF122	8.78E-03	Ethylene signaling pathway, Transcription, Transcription regulation
II-B	OOPS	1	2.3e-153	24	Q8L7W9	abi4	1.26E-02	Abscisic acid-activated signaling pathway, seed development, transcription
II-B	OOPS	2	2.7e-130	24	Q8L9Y3	ARR14	6.35E-03	Cytokinin-activated signaling pathway, transcription, two-component regulatory system, activate some type-A response regulators in response to cytokinins
II-B	OOPS	2	2.7e-130	24	Q9FXD6	ARR11	2.91E-03	Cytokinin-activated signaling pathway, regulation of root meristem growth, response to cytokinin, transcription, two-component regulatory system
II-B	OOPS	2	2.7e-130	24	Q9ZWJ9	ARR2	1.31E-02	Cellular response to cytokinin stimulus, cytokinin-activated signaling pathway, ethylene-activated signaling pathway, leaf senescence, regulation of root meristem, regulation of seed growth, regulation of stomatal movement, response to cytokinin, response to ethylene, root development, transcription
II-B	OOPS	3	5.2e-103	24	A9TN45	Predicted protein	9.55E-04	
II-B	OOPS	3	5.2e-103	24	P92948	CDC5	5.01E-04	Cell cycle, cell differentiation, defense response signaling pathway, defense response to bacterium, defense response to fungus, DNA repair, innate immune response, mRNA processing, regulation of transcription, regulation of transcription from RNA polymerase II promoter, RNA splicing, transcription
II-B	OOPS	3	5.2e-103	24	Q9LTC7	BHLH34	5.37E-04	Regulation of transcription, transcription
II-B	OOPS	4	3.0e-088	24	F4JRB0	HHO5	6.46E-03	Floral organ formation, negative regulation of gene expression, regulation of transcription, specification of plant organ identity, transcription
II-B	OOPS	4	3.0e-088	24	Q9LVR0	ATHB-53	3.57E-03	Regulation of auxin/cytokinin signaling during root development, response to auxin, root development, transcription
II-B	OOPS	4	3.0e-088	24	Q9ZPX0	GATA20	2.84E-03	Transcription, Transcription regulation; Transcriptional regulator that specifically binds 5'-GATA-3' or 5'-GAT-3' motifs within gene promoters
II-B	OOPS	5	1.1e-074	24	Q75HX9	Predicted protein	1.67E-05	
II-B	OOPS	5	1.1e-074	24	Q9M7Q3	ABF3	1.67E-05	Abscisic-acid activated signaling pathway, response to abscisic acid, response to salt stress, response to water deprivation, transcription
II-B	OOPS	5	1.1e-074	24	Q9SJN0	ABI5	1.54E-05	Abscisic-acid activated signaling pathway, negative regulation of seed germination, positive regulation of transcription, response to abscisic acid, response to chitin, response to gibberellin, response to salt stress, response to water deprevation, seed development, seed germination, sugar mediated signaling pathway, transcirption

III-B	OOPS	1	####	16	Q02994	myb.Ph3	3.80E-03	DNA binding
III-B	OOPS	1	####	16	Q8VZS3	HHO2	4.83E-03	DNA binding, DNA binding transcription factor activity, phosphate ion homeostasis, regulation of transcription, DNA-templated, response to abscisic acid, transcription, DNA-templated
III-B	OOPS	1	####	16	Q9FX84	HHO6	8.38E-03	DNA binding, DNA binding transcription factor activity, regulation of transcription, DNA-templated, transcription, DNA-templated
III-B	OOPS	2	####	16	Q6NKN9	BHLH74	4.11E-03	DNA binding, DNA binding transcription factor activity, protein dimerization activity, regulation of growth, regulation of transcription, DNA-templated, response to blue light, transcription, DNA-templated
III-B	OOPS	2	####	16	Q01593	ABI3	6.02E-03	DNA binding, DNA binding transcription factor activity, transcription factor activity, RNA polymerase II transcription factor binding, abscisic acid-activated signalling pathway, embryo development ending in seed dormancy, mitochondria-nucleus signalling pathway, plastid organization, positive regulation of transcription, DNA-templated, response to abscisic acid, response to auxin, transcription, DNA-templated
III-B	OOPS	2	####	16	Q5YGP8	PLT1	1.52E-02	DNA binding, DNA binding transcription factor activity, auxin-activated signaling pathway, ethylene-activated signaling pathway, pattern specification process, root development, root meristem growth, stem cell population maintenance, telomere maintenance, transcription, DNA-templated
III-B	OOPS	3	####	16	Q49550	DOF4.5	5.85E-03	DNA binding, DNA binding transcription factor activity, metal ion binding, regulation of transcription, DNA-templated, transcription, DNA-templated
III-B	OOPS	3	####	16	Q9SQQ6	NAC046	7.18E-03	DNA binding, DNA binding transcription factor activity, positive regulation of chlorophyll catabolic process, positive regulation of leaf senescence, transcription, DNA-templated
III-B	OOPS	3	####	16	Q9LS50	NAC058	1.18E-02	DNA binding, DNA binding transcription factor activity, transcription, DNA-templated
III-B	OOPS	4	####	16	A9RZ73	PHYPADRAFT_72483	2.07E-03	protein dimerization activity
III-B	OOPS	4	####	16	Q9CAA4	BIM2	3.31E-03	DNA binding transcription factor activity, protein dimerization activity, sequence-specific DNA binding, transcription, DNA-templated
III-B	OOPS	4	####	16	Q49404	BEH3	3.91E-03	DNA binding, DNA binding transcription factor activity, brassinosteroid mediated signaling pathway, regulation of transcription, DNA-templated, transcription, DNA-templated
III-B	OOPS	5	####	16	I3NN78	ZF2	2.74E-04	nucleic acid binding
III-B	OOPS	5	####	16	B9GPL8	POPTR_0002s00440g	1.52E-03	DNA binding, metal ion binding
III-B	OOPS	5	####	16	Q9SMX9	SPL1	1.92E-03	DNA binding, DNA binding transcription factor activity, metal ion binding, regulation of transcription, DNA-templated, transcription, DNA-templated
V-B	OOPS	1	####	20	Q1PFR7	LEC2	2.58E-04	DNA binding, DNA binding transcription factor activity, embryo development ending in seed dormancy, positive regulation of auxin biosynthetic process, positive regulation of transcription, DNA-templated, seed maturation, seed oilbody biogenesis, somatic embryogenesis, transcription, DNA-templated
V-B	OOPS	1	####	20	Q9FY74	CAMTA1	1.23E-03	calmodulin binding, sequence-specific DNA binding, transcriptional activatory activity, RNA polymerase II proximal promoter sequence-specific DNA binding, positive regulation of transcription, DNA-templated, positive regulation of transcription from RNA polymerase II promoter, response to auxin, response to freezing, response to water deprivation
V-B	OOPS	1	####	20	Q9LIE5	FHY3	1.41E-03	DNA binding transcription factor activity, zinc ion binding, circadian rhythm, far-red light signaling pathway, positive regulation of circadian rhythm, positive regulation of transcription, DNA-templated, red, far-red light phototransduction, red or far-red light signaling pathway, response to far red light, transcription, DNA-templated
V-B	OOPS	2	####	20	Q5DW98	PEND	7.67E-03	
V-B	OOPS	2	####	20	Q9FM03	DOF5.6	1.57E-02	DNA binding, DNA binding transcription factor activity, metal ion binding, phloem or xylem histogenesis, positive regulation of transcription, DNA-templated, procambium histogenesis, regulation of transcription, DNA-templated, transcription, DNA-templated
V-B	OOPS	2	####	20	Q84TE9	DOF5.3	1.67E-02	DNA binding transcription factor activity, metal ion binding, transcription regulatory region DNA binding, regulation of transcription, DNA-templated, root development, transcription, DNA-templated
V-B	OOPS	3	####	20	O82155	DOF1.7	1.64E-06	DNA binding transcription factor activity, metal ion binding, transcription regulatory region DNA binding, regulation of transcription, DNA-templated, response to chitin, transcription, DNA-templated
V-B	OOPS	3	####	20	Q9SEZ3	CDF5	3.75E-06	DNA binding, DNA binding transcription factor activity, metal ion binding, flower development, negative regulation of long-day photoperiodism, flowering, negative regulation of short-day photoperiodism, flowering, regulation of transcription, DNA-templated, transcription, DNA-templated
V-B	OOPS	3	####	20	Q9FGD6	DOF5.8	3.85E-06	DNA binding transcription factor activity, metal ion binding, transcription regulatory region sequence-specific DNA binding, regulation of transcription, DNA-templated, transcription, DNA-templated
V-B	OOPS	4	####	20			9.12E-10	
V-B	OOPS	4	####	20	Q9SKD0	BPC1	1.40E-07	DNA binding, sequence-specific DNA binding, regulation of developmental process, regulation of transcription, DNA-templated, response to ethylene, transcription, DNA-templated
V-B	OOPS	4	####	20	Q8L999	BPC6	1.78E-07	protein homodimerization activity, sequence-specific DNA binding, regulation of transcription, DNA-templated, response to ethylene, transcription, DNA-templated
V-B	OOPS	5	####	20	Q9FGV1	ARF8	1.58E-04	DNA binding transcription factor activity, sequence-specific DNA binding, auxin-activated signaling pathway, flower development, response to auxin, transcription, DNA-templated
V-B	OOPS	5	####	20	Q9SGJ6	DREB1E	4.27E-04	DNA binding, DNA binding transcription factor activity, regulation of gibberellin biosynthetic process, regulation of timing of transition from vegetative to reproductive phase, transcription, DNA-templated
V-B	OOPS	5	####	20	Q94JM3	ARF2	5.19E-04	DNA binding transcription factor activity, sequence-specific DNA binding, auxin-activated signaling pathway, floral organ abscission, fruit dehiscence, leaf senescence, negative regulation of cell proliferation, negative regulation of transcription, DNA-templated, plant ovule development, positive regulation of flower development, positive regulation of potassium ion import, response to abscisic acid, transcription, DNA-templated

Un-B	OOPS	1	####	19	Q9M2U1	DOF3.6	1.22E-10	DNA binding, metal ion binding, regulation of transcription, DNA-templated, transcription, DNA-templated
Un-B	OOPS	1	####	19	Q9LZ56	DOF5.1	5.57E-09	DNA binding, DNA binding transcription factor activity, metal ion binding, regulation of transcription, DNA-templated, transcription, DNA-templated
Un-B	OOPS	1	####	19	Q9SEZ3	CDF5	1.22E-08	DNA binding, DNA binding transcription factor activity, metal ion binding, flower development, negative regulation of long-day photoperiodism, flowering, negative regulation of short-day photoperiodism, flowering, regulation of transcription, DNA-template
Un-B	OOPS	2	####	19	F4JRB0	HHO5	3.93E-04	DNA binding transcription factor activity, transcription regulatory region DNA binding, floral organ formation, negative regulation of gene expression, regulation of transcription, DNA-templated, specification of plant organ identity, transcription, DNA-template
Un-B	OOPS	2	####	19	F4JRB0	HHO6	1.85E-03	DNA binding transcription factor activity, transcription regulatory region DNA binding, floral organ formation, negative regulation of gene expression, regulation of transcription, DNA-templated, specification of plant organ identity, transcription, DNA-template
Un-B	OOPS	2	####	19	Q9FGD6	DOF5.8	2.67E-03	DNA binding transcription factor activity, metal ion binding, transcription regulatory region sequence-specific DNA binding, regulation of transcription, DNA-templated, transcription, DNA-templated
Un-B	OOPS	3	####	19	O80917	DREB2E	2.31E-04	DNA binding transcription factor activity, sequence-specific DNA binding, transcription regulatory region DNA binding, abscisic acid-activated signaling pathway, cellular response to heat, positive regulation of transcription, DNA-templated, response to salt stress, response to water deprivation, transcription, DNA-templated
Un-B	OOPS	3	####	19	Q9ZWA6	MGP	2.86E-04	DNA binding transcription factor activity, metal ion binding, transcription regulatory region DNA binding, asymmetric cell division, regulation of transcription, DNA-templated, transcription, DNA-templated
Un-B	OOPS	3	####	19	Q9FFH3	NUC	2.86E-04	DNA binding, DNA binding transcription factor activity, metal ion binding, asymmetric cell division, leaf senescence, photoperiodism, flowering, positive regulation of transcription, DNA-templated, regulation of timing of transision from vegetative to reproductive phase, regulation of transcription, DNA-templated, transcription, DNA-templated
Un-B	OOPS	4	####	19	Q39088	DOF3.4	1.17E-03	DNA binding, DNA binding transcription factor activity, metal ion binding, transcription regulatory region DNA binding, cell wall modification, positive regulation of cell cycle, positive regulation of transcription, DNA-templated, response to auxin, response to salicylic acid, transcription, DNA-templated
Un-B	OOPS	4	####	19	Q9FGD6	DOF5.8	3.77E-03	DNA binding transcription factor activity, metal ion binding, transcription regulatory region sequence-specific DNA binding, regulation of transcription, DNA-templated, transcription, DNA-templated
Un-B	OOPS	4	####	19	P68350	DOF1.5	7.71E-03	DNA binding transcription factor activity, metal ion binding, transcription regulatory region DNA binding, regulation of transcription, DNA-templated, seed coat development, transcription, DNA-templated
Un-B	OOPS	5	####	19	Q9FKA0	NAC92	4.61E-03	DNA binding transcription factor activity, protein homodimerization activity, sequence-specific DNA binding, lateral root development, leaf senescence, positive regulation of age-related resistance, positive regulation of DNA binding transcription factor activity, positive regulation of leaf senescence, positive regulation of programmed cell death, regulation of gene expression, regulation of seed germination, response to abscisic acid, response to auxin, response to ethylene, response to hydrogen peroxide, response to oxidative stress, response to salt, response to salt stress, stress-induced premature senescence, transcription, DNA-templated
Un-B	OOPS	5	####	19	Q9SAH7	WRKY40	1.10E-02	DNA binding transcription factor activity, sequence-specific DNA binding, transcription regulatory region DNA binding, defense response to bacterium, defence response to fungus, negative regulation of transcription, DNA-templated, regulation of defense response, response to chitin, response to molecule of bacterial origin, response to salicylic acid, response to wounding, transcription, DNA-templated
Un-B	OOPS	5	####	19	Q9SJ09	WRKY59	1.21E-02	DNA binding transcription factor activity, sequence-specific DNA binding, transcription, DNA-templated
ALL-B	OOPS	1	####	95	Q9SKD0	BPC1	1.27E-13	DNA binding, sequence-specific DNA binding, regulation of developmental process, regulation of transcription, DNA-templated, response to ethylene, transcription, DNA-templated
ALL-B	OOPS	1	####	95	F4JUI3	BPC5	2.00E-12	sequence-specific DNA binding, regulation of transcription, DNA-templated, response to ethylene, transcription, DNA-templated
ALL-B	OOPS	1	####	95	Q8L999	BPC6	3.25E-12	protein homodimerization activity, sequence-specific DNA binding, regulation of transcription, DNA-templated, response to ethylene, transcription, DNA-templated
ALL-B	OOPS	2	####	95	Q9FGD6	DOF5.8	3.58E-11	DNA binding transcription factor activity, metal ion binding, transcription regulatory region sequence-specific DNA binding, regulation of transcription, DNA-templated, transcription, DNA-templated
ALL-B	OOPS	2	####	95	Q9LZ56	DOF5.1	3.80E-11	DNA binding, DNA binding transcription factor activity, metal ion binding, regulation of transcription, DNA-templated, transcription, DNA-templated
ALL-B	OOPS	2	####	95	Q9M2U1	DOF3.6	5.53E-11	DNA binding, metal ion binding, regulation of transcription, DNA-templated, transcription, DNA-templated
ALL-B	OOPS	3	####	95				
ALL-B	OOPS	3	####	95				
ALL-B	OOPS	3	####	95				
ALL-B	OOPS	4	####	95	Q9C8P8	BHLH80	1.36E-03	core promoter sequence-specific DNA binding, DNA binding transcription factor activity, protein dimerization activity, transcriptional activator activity, RNA polymerase II transcription regulatory region sequence-specific DNA binding, cuticle development, regulation of transcription, DNA-templated, transcription from RNA polymerase II promoter
ALL-B	OOPS	4	####	95	Q94JL3	BHLH112	1.66E-03	DNA binding transcription factor activity, protein dimerization activity, RNA polymerase II proximal promoter sequence-specific DNA binding, sequence-specific DNA binding, transcriptional activator activity, RNA polymerase II transcription regulatory region sequence-specific DNA binding, cellular response to abscisic acid stimulus, cellular response to salt stress, cellular response to water deprivation, positive regulation of transcription, DNA-templated, regulation of proline metabolic process, regulation of reactive oxygen species metabolic process, transcription from RNA polymerase II promoter
ALL-B	OOPS	4	####	95	Q66GR3	BHLH130	2.87E-03	core promoter sequence-specific DNA binding, DNA binding transcription factor activity, protein dimerization activity, transcriptional activator activity, RNA polymerase II transcription regulatory region sequence-specific DNA binding, photoperiodism, flowering, regulation of transcription, DNA-templated, transcription from RNA polymerase II promoter
ALL-B	OOPS	5	####	95	Q9M2U1	DOF3.6	6.12E-10	DNA binding, metal ion binding, regulation of transcription, DNA-templated, transcription, DNA-templated
ALL-B	OOPS	5	####	95	Q9SEZ3	CDF5	1.49E-09	DNA binding, DNA binding transcription factor activity, metal ion binding, flower development, negative regulation of long-day photoperiodism, flowering, negative regulation of short-day photoperiodism, flowering, regulation of transcription, DNA-templated, transcription, DNA-templated
ALL-B	OOPS	5	####	95	Q9FGD6	DOF5.8	1.92E-07	DNA binding transcription factor activity, metal ion binding, transcription regulatory region sequence-specific DNA binding, regulation of transcription, DNA-templated, transcription, DNA-templated
